# A multi-gene phylogeny of Cephalopoda supports convergent morphological evolution in association with multiple habitat shifts in the marine environment

**DOI:** 10.1186/1471-2148-12-129

**Published:** 2012-07-28

**Authors:** Annie R Lindgren, Molly S Pankey, Frederick G Hochberg, Todd H Oakley

**Affiliations:** 1 Department of Ecology, Evolution, and Marine Biology, University of California, Santa Barbara, CA 93106, USA; 2 Department of Invertebrate Zoology, Santa Barbara Museum of Natural History, 2559 Puesta del Sol Rd, Santa Barbara, CA, 93105, USA; 3 Present Address: Department of Biology, Portland State University, PO Box 751, Portland, OR 97207, USA

**Keywords:** Cephalopoda, Convergent evolution, Correlated traits, Morphology, Phylogeny

## Abstract

**Background:**

The marine environment is comprised of numerous divergent organisms living under similar selective pressures, often resulting in the evolution of convergent structures such as the fusiform body shape of pelagic squids, fishes, and some marine mammals. However, little is known about the frequency of, and circumstances leading to, convergent evolution in the open ocean. Here, we present a comparative study of the molluscan class Cephalopoda, a marine group known to occupy habitats from the intertidal to the deep sea. Several lineages bear features that may coincide with a benthic or pelagic existence, making this a valuable group for testing hypotheses of correlated evolution. To test for convergence and correlation, we generate the most taxonomically comprehensive multi-gene phylogeny of cephalopods to date. We then create a character matrix of habitat type and morphological characters, which we use to infer ancestral character states and test for correlation between habitat and morphology.

**Results:**

Our study utilizes a taxonomically well-sampled phylogeny to show convergent evolution in all six morphological characters we analyzed. Three of these characters also correlate with habitat. The presence of an autogenic photophore (those relying upon autonomous enzymatic light reactions) is correlated with a pelagic habitat, while the cornea and accessory nidamental gland correlate with a benthic lifestyle. Here, we present the first statistical tests for correlation between convergent traits and habitat in cephalopods to better understand the evolutionary history of characters that are adaptive in benthic or pelagic environments, respectively.

**Discussion:**

Our study supports the hypothesis that habitat has influenced convergent evolution in the marine environment: benthic organisms tend to exhibit similar characteristics that confer protection from invasion by other benthic taxa, while pelagic organisms possess features that facilitate crypsis and communication in an environment lacking physical refuges. Features that have originated multiple times in distantly related lineages are likely adaptive for the organisms inhabiting a particular environment: studying the frequency and evolutionary history of such convergent characters can increase understanding of the underlying forces driving ecological and evolutionary transitions in the marine environment.

## Background

Large-scale shifts in habitat during evolution require populations to respond to new selective pressures, often resulting in a cascade of novel morphologies. For example, the initial land invasion by marine tetrapods required significant changes in physiology, morphology, and life history, which led to the origin of amniotic eggs, modified limbs, scales to reduce water loss, and modified lungs (e.g. [1]). Distantly related lineages that transition to similar habitats are under the same kinds of selective pressures, often resulting in convergent structures. Canonical examples include spines in plants inhabiting arid environments and flight morphologies in insects, birds, and bats. Convergence remains largely understudied in many marine invertebrates, where continued habitat shifts and diversification have led to a number of diverse lineages that possess multiple potentially convergent characters.

Potentially adaptive morphological features shared between distantly related organisms inhabiting similar environments provide an opportunity to test for correlation between morphology and habitat to provide new hypotheses about the factors influencing evolution and diversification. For example, stickleback fish have long been studied for their numerous instances of convergent characters such as body shape, pelvis morphology, skeletal armor, and mating preferences, associated with transitions from marine to freshwater benthic and limnetic habitats (e.g. [2-5]). In sticklebacks, recent work by Jones et al. [6] identified a core set of genome-wide loci that are associated with the transition from marine to freshwater habitats, shedding light on the underlying genes that are responding to and driving adaptive evolution. In other marine taxa, numerous cases of convergent evolution have been found, including: very similar antifreeze glycoproteins that occur in phylogenetically distinct Arctic and Antarctic fishes [7], the presence of marine bioluminescence in almost all marine invertebrate phyla (e.g. [8-10]), and the thunniform body shape seen in whales, ichthyosaurs, and several species of large pelagic fishes [11]. However, in cephalopods, studies that examine the potential for correlation between habitat and morphology have been largely limited to physiology. Childress [12] argued that many of the observed relationships between metabolism and depth in oceanic squids were the result of adaptation rather than phylogenetic history, because similar metabolic rates consistently coincide with depth, even across distantly related taxa. Seibel and Carlini [13] tested this hypothesis in a phylogenetic context using a molecular-based phylogeny, and found that correlation between changes in metabolism and depth existed in the pelagic squids.

The marine environment can be broadly divided into two macrohabitats, each promoting a suite of adaptations in the inhabitants. The largely two-dimensional demersal habitats include both the benthic and benthopelagic habitat, where organisms live on or just above the bottom, respectively. Demersal habitats are dominated by animals with defensive techniques that do not require extended periods of rapid movement, such as releasing ink and then jetting away, burrowing in sediment or emitting toxic substances. Many demersal fishes and cephalopods commonly utilize cryptic behaviors that involve changing body coloration, texture, and/or shape to avoid predation (e.g. [14,15]). In contrast, the three-dimensional pelagic environment is dominated by animals that are capable of rapid movement, that often have body plans capable of responding to stimuli from several directions, and that use cryptic techniques such as transparency, reflection, and ventral bioluminescence (e.g. [14,16]) to allow for improved predation, communication, and predator avoidance. Transitions between demersal and pelagic habitats during evolution should promote shifts in suites of morphological features, a hypothesis that can be examined with phylogenetic comparative analyses on lineages living in both habitats.

Modern cephalopods occupy nearly every habitat of the marine environment from the rocky intertidal to the deep sea, making them an excellent group in which to test for convergent evolution. The two most speciose lineages, Decapodiformes (pelagic squids, bobtail squids, and cuttlefishes) and Octopodiformes (octopuses and *Vampyroteuthis infernalis*) are each comprised of demersal and pelagic taxa. Although the two cephalopod lineages are morphologically very distinct, some features seem to appear consistently in species inhabiting similar environments. Demersal taxa generally tend to have a more bulbous body, corneas covering the eye, and exhibit some of the most advanced crypsis seen in the marine environment (e.g. [14,17-19]). Pelagic cephalopods often possess photophores for communication and crypsis, fins to help with propulsion, the ability to become almost entirely transparent, and a flexible internal shell for stability. However, there are exceptions to these trends. For example, the squid family Loliginidae, the most common group in the lineage Myopsida (Decapodiformes) is an important fisheries group that lives in coastal waters and lays its eggs in the benthic environment, but feeds in the water column (e.g. [20]). Loliginids have a streamlined, muscular body morphology and internal shell similar to many pelagic squids, while bearing corneas reminiscent of benthic squids, bacteriogenic photophores (in some species), and accessory reproductive structures found largely in benthic species.

To study the phylogenetic and environmental forces driving character evolution requires a well-sampled phylogeny. In cephalopods, molecular data sets have provided many new phylogenetic hypotheses, but have either had limited taxon sampling (e.g. [13,21]) or recovered limited resolution for some major extant lineages [22-25]. Here, we assemble previously published data from up to 10 genes for over 400 cephalopod taxa (representing 42 of 47 known families) to create the most comprehensive molecular analysis of cephalopod phylogeny to date, and subsequently use the phylogeny to test the hypothesis that habitat shifts drive convergent evolution in cephalopods. We first use the phylogeny to reconstruct ancestral character states for several characters whose evolutionary history remain elusive: the accessory nidamental gland, a specialized structure that provides antimicrobial protection to eggs laid on the ocean floor; the branchial canal, a structure often thought to be a synapomorphy uniting bobtail squids, cuttlefishes, and loliginids; the cornea, which covers the eye in many demersal taxa and whose status as a synapomorphy remains unclear; the presence of the right oviduct, another important potential synapomorphy for major clades; and photophores, which occur in many lineages of Cephalopoda (e.g. [22,26,27]). We then are able to test whether any of these characters are correlated with either demersal or pelagic lifestyles, thus providing new information regarding convergent evolution in the marine environment.

## Results

Based on extensive new phylogenetic analyses including most available cephalopod molecular data, we find that transitions between demersal and pelagic habitats were common during the evolutionary history of cephalopods. We report strong evidence for convergent evolution of multiple phenotypic traits and correlated evolution between habitat and some of these traits, including accessory nidamental glands (ANG), bioluminescent photophores, and corneas. Below, we detail our results relating to new phylogenetic analyses, convergent trait evolution, habitat shifts, and correlations between habitat and morphology. These results are indicative of convergent adaptations to demersal or pelagic marine habitats in cephalopods.

### A well-sampled phylogeny

**Figure 1:**
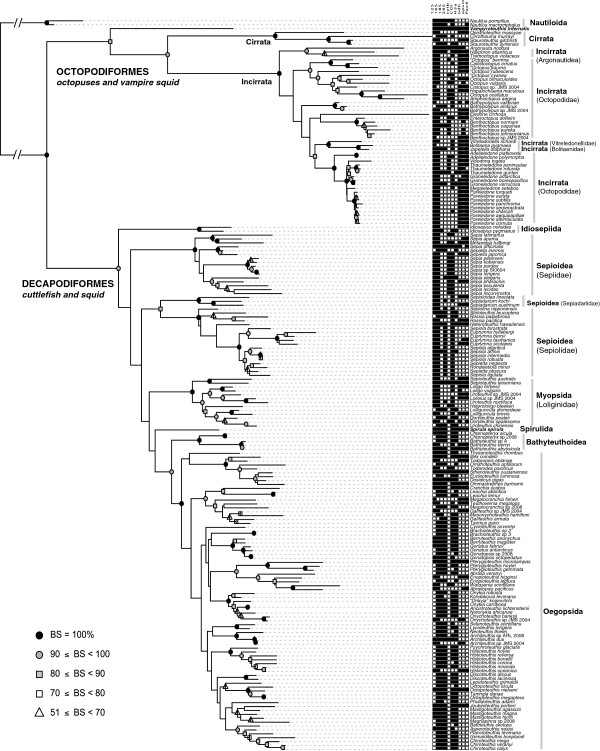
**Maximum-likelihood topology of the dataset containing taxa represented by more than three loci.** Support values of nodes were generated via 1000 bootstrap replicates and nodes are labeled as follows: black circle (100%), grey circle (90-99%), grey square (80-89%), white square (70-79%), white triangle (51-69%). Nodes lacking symbols represent bipartitions recovered in less than 50% of the bootstrapped topologies. The presence of sequence data for a particular locus is indicated by a black square following the terminals.

Our phylogenetic analyses include the highest degree of taxonomic diversity of any published study on cephalopods to date (42 of 47 coleoid families). This phylogeny allows explicit testing of hypotheses of convergent adaptations of cephalopods to different marine environments. Results from phylogenetic analysis of the reduced 188-taxon dataset (Figure 1) confirms monophyly for almost all major orders and for all families studied except Octopodidae, which is a highly morphologically divergent group with numerous species complexes [28]. The sepioid orders at the base of the decapodiform clade did not form a monophyletic group, and the position of Loliginidae was ambiguous. Such ambiguity may be due to factors such as substitution saturation and/or rate heterogeneity: we found saturation in several mitochondrial genes, more pronounced in decapodiforms than octopods (Additional file 1 Figures S5-S14), which could hinder attempts to resolve ancient, rapid radiations [29]. We accounted for this phylogenetic uncertainty in tests of character evolution by analyzing all hypotheses on multiple phylogenies estimated from bootstrapped, pseudoreplicated data sets. Despite phylogenetic uncertainty, we uncovered clear signals of convergent and correlated character evolution. 

### Multiple transitions between demersal and pelagic habitats

**Figure 2:**
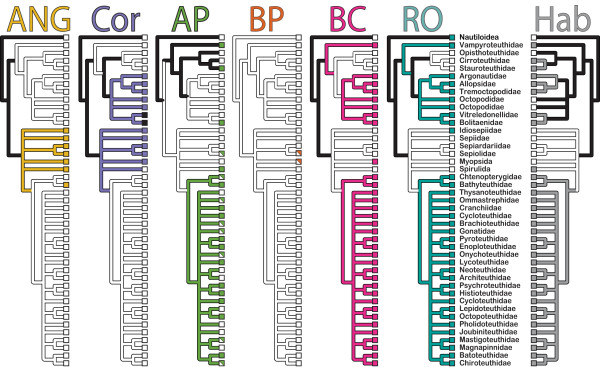
**Character state analyses on the 188-taxon tree summarized at the family-level.** Ancestral states for habitat type and six morphological traits were reconstructed and tested for correlation on each of 1000 bootstrap replicates of our 188-taxon dataset. Extant morphological state presence for the six characters examined is shown in tip boxes as follows: accessory nidamental gland (yellow); branchial canal (pink); corneas (violet); right oviduct (teal); autogenic photophore (green); and bacteriogenic photophore (orange). The taxa found in pelagic habitats are shaded in grey while demersal taxa are white in the tree at right. More detail on character states and coding can be found in Additional file 2. Internal branches are shaded with: trait color, when ASR tests significantly favored the presence of the trait; white, when ASR tests significantly favored an absent state; black, when neither state fit significantly better.

Our phylogenetic analyses support multiple habitat transitions during the history of cephalopods, which allows us to test whether convergent phenotypes are associated with such shifts. Habitat reconstruction across the 1000 bootstraps of our 188-taxon tree (Figure 2, condensed to family, see also Additional file 1 Figure S2), indicates that transitions to the pelagic realm are relatively common, while reciprocal transitions to demersal habitats have been rare. The lack of viable outgroup taxa, coupled with rapid evolution, which erases deep history, led to ancestral state analyses uninformative about the habitat state of the ancestor to Cephalopoda. Nevertheless, the deep ancestor of octopuses, true squids, loliginids, bobtail squids and cuttlefish (Coleoidea) received more support for a demersal state, with 19% of bootstrap trees recovering significantly greater proportional likelihood at this node. In contrast, we recover a pelagic existence for the ancestor of the octopuses and *Vampyroteuthis* (Octopodiformes), with 14% of bootstrapped topologies yielding significantly higher proportional likelihoods for this outcome compared to zero in favor of a demersal ancestor of Octopodiformes, a finding contradictory to previous hypotheses [30]. Young et al. [30] proposed that the immediate ancestor of *Vampyroteuthis* had an oral orientation that pre-adapted it for settling on the ocean floor, indicating a benthic ancestor. The subsequent transition to a demersal lifestyle in the cirrate/incirrate ancestor (33% of bootstrapped trees favoring the demersal state compared to zero favoring a pelagic state) represents the sole case of a pelagic-to- demersal transition that we recovered. The ancestral cirrate lineage also underwent a secondary transition to the pelagos, a scenario consistent with previous hypotheses based on morphology [30]. The ancestral decapodiform squid was likely demersal in nature, with recent transitions to the pelagic realm in some sepiolid lineages as well as an ancient transition to the pelagic environment occurring in the ancestor of the pelagic orders Oegopsida and Bathyteuthoidea, the latter finding reinforced by fossil data [31]. 

### Convergent evolution in Cephalopoda

Ancestral state reconstruction of six morphological characters on the 188-taxon dataset suggests that convergent evolution is a common phenomenon across cephalopods, as all six morphological characters we examined showed evidence of convergence (collapsed to family in Figure 2, see Figure 1 for complete topology). However, these characters were chosen based on a hypothesis that habitat has shaped their evolution, and therefore may not represent overall rates of convergent evolution in cephalopods.

#### ANG

An accessory nidamental gland (ANG) is present in squid families Idiosepiidae, Sepiolidae, Sepiidae, Spirulidae, Loliginidae, and Chtenopterygidae (e.g. [20,32]). The ANG is present solely in females where it is thought to provide an additional protective coating of mucous on eggs. Female nautiloids and octopodiforms lack the accessory nidamental gland. Several octopods, as well as some oegopsids, lack a primary nidamental gland entirely, instead relying on oviducal glands on the oviducts that aid in egg secretion. Our reconstruction analysis finds the ancestral female decapodiform squid likely possessed an ANG (76% of bootstraps yielding significantly higher likelihoods for the present state). Although gain/loss rates for this trait are relatively low (not shown), losses likely occurred following transitions to pelagic lifestyles in the squid lineage.

#### Corneas

Our results are consistent with the hypothesis that the cornea is convergent between squids and octopods, a finding also supported by developmental data [33]. The decapodiform ancestor likely possessed a one-part cornea, which was lost with the transition to a pelagic lifestyle. In the octopod lineage, the basal pelagic groups *Vampyroteuthis* and Cirrata both lack a cornea. In the incirrate octopods, two pelagic clades possess reduced corneas or have lost corneas altogether, while all benthic octopods possess a fully formed two-part cornea.

#### Autogenic and bacteriogenic photophores

Bioluminescent organs, or photophores, have been acquired numerous times throughout Cephalopoda and rates of gain and loss suggest a very dynamic history (Figure 2). Of all characters tested, autogenic photophores are the sole trait for which asymmetrical rates of gain and loss significantly improve the fit of the character model. Photophores appear to be lost at similarly moderate rates regardless of habitat, but show significant rates of gain in pelagic lineages, which is echoed by the structural diversity seen in these organs. Autogenic organs were likely present in the ancestor of the bathyteuthoids and oegopsids. If Spirulidae is sister to Bathyteuthoidea + Oegopsida, then autogenic bioluminescence may be synapomorphic among all three lineages. However, if Spirulidae is more closely related to sepioids, then we predict that autogenic photophores evolved separately in Spirulidae and in Bathyteuthoidea + Oegopsida. Autogenic bioluminescence in the Octopodiformes likely originated separately in *Vampyroteuthis*, the cirrate octopod genus *Stauroteuthis*, and in both genera (*Bolitaena* and *Japatella)* of the incirrate octopod family Bolitaenidae. While both forms of bioluminescence were included in subsequent habitat correlation analysis, bacteriogenic organs are not known from pelagic taxa.

Bacteriogenic photophores are known only from some demersal species from both sepiolid and loliginid lineages. Bacteriogenic photophores (those harboring symbiotic bacteria from the family Vibrionaceae, e.g. [34-36]) occur in the demersal Loliginidae and Sepiolidae clades and appear to have evolved separately. Ancestral reconstruction supports the hypothesis of convergence for this character, as the MRCA of sepiolids and loliginids is predicted to have lacked a photophore.

#### Branchial canal

The branchial canal is present at the base of the gill lamellae of *Vampyroteuthis,* the incirrate octopods, Loliginidae, Bathyteuthoidea, and Oegopsida. Our findings suggest that this character is ancestral in the Octopodiformes (lost in the cirrates) and evolved convergently in the decapodiforms.

#### Right oviduct

Statistical analyses indicate that the right oviduct was likely present in the octopodiform ancestor and subsequently lost in the ancestral cirrate and gained independently within the decapodiform lineage. Ancestral state reconstruction also suggests that the right oviduct may have evolved convergently in the oegopsids and in Idiosepiidae, where is it highly reduced [27].

### Correlation between phenotypes and marine habitats

Of the six characters we examined, three show correlation with marine habitat, supporting hypotheses that these characters are adaptive. A demersal lifestyle is correlated with the presence of accessory nidamental glands in females (p = 0.0147) and marginally correlated with the presence of corneas (p = 0.0747). In addition, we find a significant correlation between pelagic lifestyle and autogenic photophore presence (p = 0.0089). In contrast, branchial canal, right oviduct, and bacteriogenic photophores show no correlation with habitat type (Table 1).

**Table 1:** Results of Pagel’s correlation analysis

**Character correlation tested**	**Independent model (LnL)**	**Dependent model (LnL)**	**Model LnL difference**	**P(X**_**df=4**_^**2**^**)**
Habitat x ANG	−37.312	−31.123	−6.190	**0.0147**
Habitat x Cornea	−37.497	−33.551	−4.253	**0.0747**
Habitat x Autogenic-photophore	−90.043	−79.445	10.598	**0.0003**
Habitat x Bacteriogenic-photophore	−51.143	−47.450	3.693	0.1169
Habitat x Branchial canal	−38.952	−38.915	−0.075	0.9973
Habitat x Right oviduct	−37.813	−34.861	−2.952	0.2062

Correlations between character transitions were evaluated against 1000 bootstrap ML topologies to correct for phylogenetic bias. To carry out correlations in BayesTraits, all characters were coded as binary (e.g., presence/absence). Median likelihood scores under each model are shown, although likelihood differences were evaluated by pairwise comparison for each bootstrap tree.

## Discussion

Convergence occurs frequently in the marine environment. In cases where distantly related taxa inhabit similar, or sympatric habitats, the presence of similar characteristics may be indicative of adaptation: in the open ocean, features such as a fusiform (or thunniform) body and silvering are present in a breadth of organisms to aid in hunting and predator avoidance (e.g. [16]). However, in cases where taxa are more closely related, it can be difficult to distinguish between phylogenetic and environmental influences. Comparative methods enable scientists to infer adaptations in the context of phylogeny, which provides insight into the interplay between phenotype and environment (e.g. [37-41]) although some argue that these methods only take into account current usage and therefore cannot accurately represent historical processes [42,43]. Martins [39] summarized the two general approaches that incorporate phylogeny when attempting to classify characters as adaptations: reconstructing ancestral character states (e.g. [42,43] and testing for statistical correlation between habitat and morphology in a phylogenetic context (e.g. [38]). Both approaches have pitfalls: a large number of taxa are required for calculating statistical correlation, and a thorough understanding of single traits is required to reconstruct their ancestral states. Here, we overcome both of these challenges with a new, much larger, phylogenetic data set and a thorough examination of a small number of morphological characters, illustrating that cephalopods are an attractive group for testing hypotheses of convergence and adaptation in the marine environment. Modern cephalopods, not unlike extinct ammonoids [44] likely have also undergone several major habitat shifts and presently occupy a plethora of habitats, ranging from the intertidal, to the open ocean, to the hydrothermal vents. Transitions to such a diverse array of niches are echoed in the presence of a myriad of morphological characteristics, many of which appear to be convergent. Here, we explicitly tested hypotheses of morphological convergence in taxa living in similar marine environments, which required large-scale reconstruction of cephalopod phylogeny.

### Phylogenetic relationships

Historically, the high rates of evolution evident in cephalopods coupled with their radiation into numerous habitats has confounded our ability to recover consistent phylogenetic relationships using either morphological [22,27,45,46] or molecular [22-24,47-53] data. Our analyses include the most comprehensive taxon sampling to date and provide a more rigorous assessment of phylogenetic relationships. Here, we recover the two major coleoid lineages with higher support values than in any previous study: Octopodiformes (all octopuses and *Vampyroteuthis*) and Decapodiformes (bobtail squids, cuttlefishes, pygmy squids, and true squids), as well as several lower-level relationships, discussed below.

Within Octopodiformes, we recovered monophyly for the higher-level groups Cirrata (the pelagic, finned octopods) and Incirrata (the benthic and pelagic octopods that lack fins), the pelagic octopod super-family Argonautoidea, and the pelagic octopod families Vitrellidonellidae and Bolitaenidae. The benthic family Octopodidae, which contains the majority of octopod species, was rendered non-monophyletic due to the internal placement of the pelagic vitrelledonellid + bolitaenid clade. The lack of monophyly for the family Octopodidae is not surprising. Although some morphological studies have suggested the Octopodidae to be monophyletic (e.g. [46]), current molecular and combined analyses have demonstrated that neither the family Octopodidae, nor the genus *Octopus,* are monophyletic (e.g. [48,54]), suggesting a very dynamic evolutionary history. Our study finds support for the division of Octopodidae into several separate families, although more rigorous morphological and molecular examination is warranted before taxonomic changes can be made.

Within Decapodiformes, we recovered several important relationships, such as the monophyly of the pelagic lineages, Bathyteuthoidea, Oegopsida, Spirulida, and Myopsida, while the benthic lineages formed several distinct clades. While support values for the clade containing Bathyteuthoidea and Oegopsida are higher than in any previous study, the position of Spirulida (the ram’s horn squid) and Myopsida has low bootstrap support (56%). The benthic pygmy squids (Idiosepiidae) was the sister group to the rest of the Decapodiformes (with low support), while the benthic bobtail squids (Sepiolidae and Sepiadariidae) and cuttlefishes (Sepiidae) formed separate clades. Previous morphological analyses suggest that the sepioid orders are likely monophyletic (e.g. [26,27,55,56]), but molecular data has been unable to recover consistent monophyly (e.g. [22-24,47,49,51,53,57]). The lack of resolution at the base of the decapodiform clade could also be due to the lack of a close living outgroup. The two coleoid lineages, Octopodiformes and Decapodiformes, are known to exhibit a substantial degree of genetic differentiation in their ribosomal genes [58,59], which likely causes difficulties in rooting the Decapodiformes clade. The ambiguity in terms of clade placement among the benthic squids and Myopsida is not a new phenomenon. Naef [26] discussed the difficulties in placing myopsids relative to other squids, eventually placing loliginids (Myopsida) as the sister group to the pelagic oegopsids in the order Teuthoidea, citing characters such as the branchial canal, and gladius (the modified internal shell) as potential synapomorphies. Previous analyses have been ambiguous; some have placed myopsids near the sepioids (e.g. [22,55]) and others as sister to the oegopsids/bathyteuthoids [24,25]. It is possible that increased gene or taxon sampling could remedy the ambiguity over the placement of the loliginids. In particular, the inclusion of a second myopsid family, Australiteuthidae, which has yet remained uncollected for molecular data, could provide additional insight.

### Character correlation and habitat

Habitat states were assigned based on the literature descriptions, which generally reference taxa as occupying a coastal benthic or an oceanic pelagic habitat (see the Methods section for details). Using this conservative binary coding scheme has several benefits. First, since habitat data are often limited to geographic coordinates and depth, we are able to confidently assign habitat states to more taxa in the dataset than would have been possible using multi-state habitat categories. For example, in many cases for nearshore coastal taxa the substantial data available in terms of their habitat and depth makes coding discrete states more straightforward (e.g. [60]). However, other groups were more challenging to classify, such as some cirrate octopods, which spend time in both the benthos and pelagos. Here, we condense the wide array of marine habitats into two states: neritic, bottom dwelling (demersal) and those inhabiting the water column in the open ocean (pelagic) in order to classify organisms as conservatively as possible to generate new hypotheses on character evolution and test for correlation.

Cephalopods possess numerous features that have been considered at one time or another to be convergent such as accessory nidamental glands, branchial canals, corneas, fins, internal shells, photophores, the ability to become transparent, egg brooding, and buoyancy. Several of these characteristics are not presently quantifiable due to a lack of data for living specimens or material: for example, we know very little about egg brooding in the true squids, although it has been known to occur in some pelagic taxa [61]. Transparency is of great interest to a multitude of researchers (e.g. [62]) but new insights are hindered by the technical difficulties in acquiring fresh specimens for most species. The presence of fins and internal shell morphology are also characters that may also be influenced by habitat, but were not quantified here, largely due to our inability to identify putative homologous structures. The fins of the deep-sea cirrate octopods contain “fin supports” which are one form of internal shell, while the fins of squids are muscular and are completely independent of the internal shell, making it difficult to determine independence and test for correlation, particularly in octopods. Furthermore, it has been extremely difficult to divide the internal shell into distinctive character states as many different morphologies exist: oegopsid, bathyteuthoid, and loliginid squid all have an internal shell in the form of a gladius, but its evolutionary history is unknown. Cuttlefishes have a cuttlebone, and sepiolids have what may be a highly reduced gladius in some species, but again, more work is needed to determine if separate character states are needed. The internal shell and fins represent a common problem with linking morphological and molecular data in cephalopods: one has to have a reasonable hypothesis of homology *a priori*. Here, we chose to focus on six characters whose morphology could be divided into distinct states with a substantial degree of confidence based on the authors’ examination of specimens and the literature. Of the six characters we examined, three correlate to habitat: accessory nidamental glands, corneas, and photophores. These characters are correlated when analyzed under alternate, pseudoreplicated topologies, indicating the results are robust to ambiguities in our phylogeny. Each character is discussed in detail below.

#### Accessory nidamental glands (ANG)

The importance to an individual’s fitness of protecting its reproductive investment is evident in the number of behavioral, chemical, and life history strategies that have evolved. Common solutions to avoiding predation and fouling on egg masses laid in benthic habitats include maternal care (e.g. [63,64]), and chemical defenses (e.g. [65]). While female incirrate octopods typically guard and aerate egg masses they have deposited in dens, the demersal bobtail squids and cuttlefishes typically attach clutches, or festoons, of 50–400 eggs to a rocky or sandy substrate and provide no post-spawning care. The accessory nidamental gland, present in all demersal decapodiform females, secretes an additional layer of mucous on eggs prior to their attachment to the benthic substrate (e.g. [32,66-68]). The dense and diverse microbial community housed within the accessory nidamental glands and transmitted to the eggs may contain strains with antimicrobial properties, thus providing some degree of protection for vulnerable embryos against fouling organisms or egg predators [69,70]. An ANG is also present the pelagic families Chtenopterygidae and Spirulidae and although they differ slightly in position when compared to the more benthic decapodiforms, Nesis [20] indicated that this structure is likely homologous. Adult chtenopterygids are meso- to bathypelagic, but it is unclear whether their eggs are deposited on the sediment, released into the midwater, or brooded as seen in some other pelagic squid groups [61]. We find evidence that the presence of an ANG correlates with a benthic lifestyle and was present in the ancestral decapodiform squid. In contrast, female octopods (both benthic and pelagic) lack a nidamental gland and an ANG, but provide substantial post-spawning care: females lay single eggs or strings in a den or shell, and tend to them constantly by maintaining a fresh, oxygenated, water supply and staving off predators until shortly before hatching, at which point the senescent female dies quickly (e.g. [64]).

#### Corneas

Our analyses indicate that the cephalopod cornea has evolved separately in the decapodiform and octopod lineages: all demersal bobtail squids, cuttlefishes, loliginids, and octopods possess a cornea, with reductions and/or complete loss occurring in pelagic forms. Young et al. [30] argued that the cornea was convergent and likely arose in response to similar selective pressures in the benthos, a finding further supported by developmental data [33]. Corneas are thought to serve several functions, ranging from keeping the eye socket free of sediment, to providing protection against ultraviolet light for taxa living in the photic zone (e.g. [71]). While it is possible that protection from ultraviolet light may play a minor role in corneal function, many benthic taxa never occupy the photic zone but still possess a fully formed cornea, indicating that a primary protective function is more likely.

Our results indicate that the ancestor of the decapodiforms likely lived in the demersal zone and possessed a cornea, which was lost as species shifted to a pelagic lifestyle. The association between habitat and corneal morphology is less clear in the octopod lineage because members of the pelagic incirrate superfamily Argonautoidea possess a cornea. The results of the discrete analysis suggest the origins of the octopod cornea correspond to the transition to a benthic habitat in the lineage ancestral to incirrates. The second pelagic octopod clade contains two families (Bolitaenidae and Vitrellidonellidae) wherein the cornea appears to be reduced or absent, which further supports the correlation between cornea and benthic habitat. One issue with evaluating corneal morphology in this group is that the pelagic octopods are extremely gelatinous animals, who deteriorate quickly upon fixation, making it difficult to determine if the cornea is simply reduced or completely absent.

#### Photophores

Bioluminescent organs throughout animals are extremely morphologically diverse (for reviews, see [10,36,72]) and can produce light via various endogenous biochemistries, so-called ‘autogenic’ organs, or via symbiotic bacteria harbored within specialized chambers, or ‘bacteriogenic’ organs [73,74]. Similar to other organisms, both forms of photophores in cephalopods can be morphologically complex, relying on optical features such as reflectors, lenses and chromatophore-based light filters [75,76]. We find that, in general, the ability to bioluminesce autogenically is highly correlated with pelagic existence in cephalopods (p = 0.0089).

Autogenic organs are found in many pelagic species of squids, octopods and in *Vampyroteuthis* (e.g. [36]). From study of two oegopsid species, the mechanism of light production appears to involve an intrinsic luciferase photoprotein reacting with dietary coelenterazine [77-80]. The high variability in expression and morphology of autogenic photophores across and within families historically has been interpreted as evidence of separate origins [81,82]. While experimentally establishing the adaptive value of these organs remains difficult owing to the challenges of sampling and maintaining deep-sea organisms, hypotheses abound. For instance, photophores frequently are located ventral to the eyes and opaque internal organs such as the ink sac, or dispersed across the surface of the skin, presumably serving to counter-illuminate the shadow cast by organs or body and thus provide a means of camouflage against deeper-dwelling predators [36,83]. However, photophores also are found along the arms or encircling the mouth, allowing the animal to escape predation, attract or startle prey, or communicate with conspecifics [84-87]. We find that autogenic photophores were likely present in some form in the ancestor of pelagic squid, and have undergone considerable diversification in terms of anatomical positioning, morphological complexity, and emission properties. Bioluminescence in octopod lineages appears to have evolved independently in three separate lineages: in *Vampyroteuthis*, within the cirrates, and in the incirrate family Bolitaenidae. The rapid transition rates of this trait suggest that any number of evolutionary mechanisms could be in play such as: selection acting either directly or indirectly on photophore expression and/or stochastic processes varying substantially through time and across lineages. Future work investigating to what extent cephalopods might rely on shared molecular mechanisms for bioluminescence may provide clues as to how such a complex trait could exhibit rapid rates of gain and loss.

#### Branchial canal, right oviduct, bacteriogenic photophore

Of the six characters examined, the branchial canal, right oviduct and bacteriogenic photophores did not correlate with habitat, although each has evolved multiple times within cephalopods. A branchial canal is present at the base of the gill lamellae in both benthic and pelagic cephalopods (absent from Cirrata) and is used to allow for passage of seawater into the gills [27]. Traditionally, the branchial canal was thought to be a synapomorphy uniting myopsids with the pelagic squids [27], but our ancestral state reconstructions suggest this state is convergent between Decapodiformes and Octopodiformes and within Decapodiformes. A right oviduct is present in both benthic and pelagic taxa, and ancestral state analysis predicts that this trait may be convergent between squids and octopods due to a loss early in decapodiform evolution. Also, if our prediction accurately reflects evolution, then the right oviduct found in Idiosepiidae and Oegopsida is the result of convergent evolution. While previous characterizations of the right oviduct in Idiosepiidae have suggested a highly modified morphology is due to a loss of function (e.g. [88]), our reconstruction analysis offers an alternative hypothesis of homoplasy and possible gain. Bacteriogenic photophores are far less common among cephalopods than compared to autogenic organs, and are found only in some members of the more benthic families Sepiolidae and Loliginidae. Although homology among bacteriogenic organs has been debated [89], our phylogenetic analyses indicate that bacteriogenic organs evolved separately in sepiolid and loliginid clades. Bacteriogenic photophores in both lineages typically develop on the ink sac as a bilobed structure, feature prominent crystalline lenses, and can recruit and culture related species of luminescent Vibrionaceae bacteria on the organ’s epithelium lining [34,35,90]. Evidence from the sepiolid *Euprymna* indicates that bacteriogenic organs may be used for counter-illumination [91]. Understanding both the molecular and ecological factors that have given rise to such a remarkable parallel will yield tremendous insight into how recurrent phenotypes evolve.

## Conclusions

Convergent evolution occurs frequently in the marine environment, and cephalopods are no exception. Here, we identify convergent morphological characteristics in Cephalopoda, using both phylogenetic and statistical approaches and find correlation between habitat and morphology. The diverse nature of cephalopod habitat and morphology has interested researchers for hundreds of years, and yet the evolutionary history of many key characteristics has remained elusive. We provide conclusive evidence that accessory nidamental glands, corneas, and photophores are convergent and correlated with habitat. The accessory nidamental gland likely protects developing embryos from fouling microorganisms and is generally present only in decapodiforms known to attach eggs to the substrate. A cornea, one of the main components of the camera-type eye found in cephalopods and vertebrates, is present only in taxa that spend time associated with the benthos, and has evolved separately in the squid and octopod lineage albeit to serve similar purposes of protection against sediment and in some cases, ultraviolet light. Photophores, present in numerous marine phyla, are gained and lost many times throughout the cephalopod lineage, but are most commonly found in pelagic taxa.

The findings presented here in phylogenetics and character evolution create a foundation for understanding convergent evolution in the marine environment. The numerous biotic and abiotic factors that influence spatial and temporal expression for a given trait make studying convergence challenging, particularly in cephalopods where experimental studies linking function and habitat are often intractable. However, the advent of high-throughput transcriptome sequencing provides new means for generating hypotheses concerning the molecular basis for morphologically convergent evolution in experimentally challenging organisms like cephalopods. Future work utilizing these tools to study how convergent characters originate will provide us with new insight into how molecular processes such as gene duplication, gene sharing, and co-option can result in the evolution of similar morphological novelties.

## Methods

### Data assembly and alignment

Of the roughly 750 valid cephalopods species, *sensu*[92], many are rarely observed and poorly known to science, which has limited taxon sampling for molecular data. Here, all sequence data available for six nuclear genes (Histone H3a, octopine dehydrogenase, pax6, opsin, 18 S rRNA, and several regions of 28 S rRNA) and four mitochondrial genes (cytochrome *c* oxidase subunit I, cytochrome B, 12 S rRNA, and 16 S rRNA) were downloaded from GenBank and compiled into a local MySQL database yielding 409 taxonomic units with varying degrees of gene coverage (Additional file 3). Each locus was vetted for possible contamination: individual genes were aligned in Muscle under default parameters (see [93]) and analyzed in a maximum likelihood framework in RAxML v.7.0.4 [94] by generating a single tree under the GTR + GAMMA distribution model. Putative contaminants were identified as terminals with long branches relative to their sister taxa and also fell into incorrect clades (e.g. a squid in an octopus clade). These potential “contaminants” were then subjected to additional blast searches in GenBank, and the top 10 “hits” were evaluated. Any sequence identified as highly similar to another cephalopod was not considered to be a contaminant and was included in the final data matrix. Only sequences identified as a non-cephalopod (most commonly bacteria) were excluded from the analyses (see Additional file 4 for a list of identified contaminants).

Only one representative sequence for a given taxon was included for each gene in the final data matrix. When multiple copies of a gene were available on GenBank, as was often the case with COI, we included either the longest read (if length variation existed in GenBank files) or else the first read in the database. Sequences were aligned using default parameters in MAFFT [95], except for COI, which was aligned using MAFFT(−einsi) for improved accuracy. To further improve alignment accuracy, protein-coding sequences were then reverse transcribed using dna2pep.py, [96] and codon-aligned using TranslatorX [97] and RevTrans [96] to generate final DNA alignments that reflected codon positions. Lastly, we visually inspected each alignment for frame shift errors.

### Molecular phylogenetic analysis

#### Taxon versus data coverage

To test for correlation between morphology and habitat, we required a well-sampled, robust phylogeny. The largest dataset with 408 taxa may not be optimal in this case because many terminals are represented by only a few genes and may be placed incorrectly due to the heterogeneity of the data matrix rather than to phylogenetic history [98,99]. To identify the dataset that was both taxon-rich and relatively robust, nine datasets were generated and subjected to phylogenetic analyses. The largest dataset included all taxa with one or more genes present (408 taxa), while the smallest included only taxa that had at least nine loci present (17 taxa). The dataset we chose as the best topology to test for subsequent character evolution and correlation is comprised of 188 taxa represented by at least four genes (see Additional file 3 for taxa list). This dataset allows the inclusion of the greatest taxonomic diversity while maintaining robust support for all higher-level clades (greater than 75%). Furthermore, bootstrap support values for these clades evaluated on datasets with greater data matrix density (i.e., more genes, fewer taxa) were never less than 75% (Table 2). 

**Table 2:** Bootstrap support for key nodes across increasingly refined datasets

**# loci Clade**	**≥ 1**	**≥2**	**≥3**	**≥4**	**≥5**	**≥6**	**≥7**	**≥8**	**≥9**
Octopodiformes	76	9	78	**85**	2	96	--	--	--
Octopoda	80	96	87	**93**	87	--	--	--	--
Cirrata	99	100	100	**100**	100	--	--	--	--
Incirrata	94	98	95	**100**	100	100	100	100	100
Decapodiformes	98	99	99	**99**	100	95	--	--	--
Myopsida	88	89	100	**100**	100	100	--	--	--
Bathy + Oeg	60	86	99	**99**	100	73	77	82	87
Oegopsida	46	77	99	**100**	97	93	96	99	99
# taxa	409	301	242	**188**	130	61	28	18	17
% missing data	86.3	82.6	80.0	**77.3**	74.5	69.8	61.8	57.5	57.3

ML trees were inferred from datasets varying in sparseness to assess the trade-off between taxon sampling and data sampling. Datasets ranged from species-rich/data-sparse (409 species represented by at least one of 10 loci) to species-limited/data-rich (17 species contributing at least 9 of 10 loci). The column representing the optimal dataset selected for additional analyses is indicated in bold. (‘--‘indicates loss of outgroup or ingroup species required to evaluate support).

Prior to formal analyses, ModelTest [100] was used to determine which model best fit the data for each partition. Each dataset was analyzed in RAxML 7.2.8 [94] under the GTR + GAMMA model (−N 10), and support was calculated with 1,000 replicates of bootstrap resampling. To identify the dataset to use for tests of character correlation, we measured clade support and compared values (Table 2) for eight nodes commonly examined in taxonomic and molecular studies: Octopodiformes (*V. infernalis* and all octopods), Octopoda, Incirrata, Cirrata, Decapodiformes, Loliginidae, Bathyteuthoidea + Oegopsida, and Oegopsida. The data set that contained at least three representative taxa in each node and support values above 80% was used for all subsequent analyses.

#### Data partitions and analyses

Given the diverse nature of the genes in our data matrix, using the same likelihood parameter values for the entire dataset is unlikely to provide a good fit (e.g. [101,102]). To reduce the risks associated with both over- and under-parameterization, we tested partitioning schemes most likely to represent groupings of genes that may have evolved under similar conditions (Table 1). We computed log-likelihoods for each of the sixteen partition strategies using a 100 replicate maximum likelihood search (RAxML v. 7.2.8) and identified the partitioning strategy that best fit the data by comparing the log-likelihoods from each of the lesser-partitioned analyses to the most heavily partitioned (sixteen) scheme using a modified BIC test [103] as implemented by McGuire et al. [104]. This modified version of the BIC test holds the number of branch lengths constant across alternative partitioning strategies, therefore returning identical estimates to the standard BIC calculations [104].

We analyzed the final data matrix (188 taxonomic units, each represented by four or more loci) under a likelihood setting in RAxML 7.2.8 with 300 replicate tree searches under GTR + GAMMA. Each gene and the 3rd position codon sites were treated as separate partitions (16-partition scheme, Table 1). All species of *Nautilus* were listed as outgroup taxa. We then evaluated support using 1000 nonparametric bootstrap replicates (−f b) in RAxML. For comparison, the above tree search was also conducted on the complete data set (Additional file 1, Figure 1).

The partitioning strategy that best fit the data (16-part, Additional file 1 Table S1) allowed for rate parameters for the sites of each ribosomal gene as well as the non-synonymous (1st/2nd codon positions) and synonymous (3rd codon positions) sites for each coding gene to be estimated independently [105,106]. It is worth noting that the 6-partition strategy (in which nuclear, mitochondrial genes, ribosomal, 1st/2nd coding positions and 3rd codon positions are differentiated) yielded likelihoods that were close to, but still less likely than the fully partitioned scheme and the BIC evaluation confirmed that the additional parameters in the 16-part scheme significantly improved the fit of this data set.

While additional phylogenetic analyses (not shown) were carried out in PhyloBayes (v2.3) using a heterogeneous-site model (GTRCAT), our dataset failed to converge after surpassing the recommended maximum of 10,000 MCMC cycles on each of 6 chains and running for over 60 days [106]. Due to the lack of convergence in the posterior distribution, we carried out likelihood-based character analyses using our best ML tree (Figure 1) and ML bootstrap pseudoreplicates.

### Character evolution and correlation with habitat

To attempt to identify characters that have arisen via convergent evolution in response to similar selective pressures, we performed ancestral states reconstructions using our 188-taxon topology. We created a binary character matrix for habitat (benthic or pelagic) and for six features of presumed taxonomic importance (e.g. [26,27,30]) that can be putatively associated with either demersal or pelagic lifestyles [30]: accessory nidamental gland, branchial canal, cornea, right oviduct, autogenic and bacteriogenic photophores. With the exception of the accessory nidamental glands and bacteriogenic photophores, the remaining characters are present in both squid and octopod lineages, thus increasing our ability to test for correlation. Character coding was done primarily through literature, although all pelagic octopod taxa were re-examined at the Santa Barbara Museum of Natural History to confirm corneal morphology.

For the habitat data, we chose to cluster habitats into two primary categories (demersal/pelagic) to be able to conduct statistical tests of correlation, and to identify broad patterns and hypotheses that can then be further examined in on a much finer scale. All habitat data for Decapodiformes was coded from the literature [60,107]: all Octopodiformes data was coded by FGH. There is some inherent risk in subdividing the expanse of the marine environment into two discrete habitats, as each macro habitat encompasses a number of smaller ecosystems. There are numerous habitats that can fall within this spectrum, and there is some inherent risk as each macro habitat encompasses a number of smaller ecosystems. Although there are many potential subdivisions within the demersal/pelagic realms, there are still some general trends seen across the entire habitat in terms of selective pressures. For example, all taxa living in demersal environments, must to some degree, compete for space. Alternately, taxa inhabiting the pelagic realm are more impacted by the three-dimensional nature of their surroundings and must be able to respond to stimuli from numerous directions.

Likelihood-based ancestral state reconstructions were carried out in BayesTraits [108] on our 188-taxon topology for habitat type (benthic/pelagic), and for characters (present/absent): accessory nidamental gland, cornea, bacteriogenic photophore, autogenic photophore, branchial canal, and right oviduct. Prior to ASR analysis, each trait was fit to a single-transition rate and asymmetrical rate model. The best-fitting model was determined with the likelihood-ratio test.

We calculated the proportional likelihoods of either ancestral state on each of the 1000 bootstrap ML trees generated from our dataset to incorporate topological uncertainty. We specifically evaluated character state likelihoods for the high-level nodes of the best ML tree recovered in at least 90% of bootstrap replicates. For nodes that received low branch support (e.g., basal Decapodiformes), we evaluated ancestral state likelihoods for their most recent common ancestor (MRCA), such as for Oegopsida + Loliginidae. As it remains unclear whether the MRCA of Oegopsida + Loliginidae lived earlier or later than the MCRA of Sepiolida + Loliginidae, we estimated the character state likelihoods for both ancestors (Figure 2, Additional file 1 Figure S2). To determine the extent to which one character state received significantly more support over the other state at a particular node, we calculated the number of bootstrap replicates for which the ratio between the two proportional likelihood scores (e.g., L _present_/L _absent_) was greater than 7.4 [109]. In addition, for each node of interest, we calculated the likelihoods across 1000 bootstrap trees fixed for either state using BayesTraits’ ‘fossil’ command. State models which yielded log-likelihood scores having two or more units higher than the other state model are regarded as a significantly better fit [110,111].

To test whether certain cephalopod characteristics are correlated with demersal or pelagic existence, we used Pagel’s correlations as implemented in BayesTraits [108] on our 188-taxon topology. We tested for the correlation between habitat and each binary trait by comparing the fit of a dependent and independent character model on bootstrapped ML trees. Under the independent model, the number and timing of gains and losses for each trait (e.g., habitat and the morphological trait) occur independently, whereas the dependent model provides additional parameters that affect the probability of a character’s transitioning on a branch depending on the state of the other character. To account for the phylogenetic uncertainty in our dataset, we fit these models for each character set on 1000 bootstrap ML topologies. We then calculated median log-likelihoods for each model and used the log-ratio test to determine which characters are significantly correlated.

## Competing interests

The authors declare that they have no competing interests.

## Authors’ contributions

ARL, MSP, and THO conceived of the study, participated in its design and coordination, and helped to draft the manuscript. ARL and MSP generated the molecular database from GenBank and vetted it for possible contaminants. MSP and THO wrote the scripts used in the data analysis pipeline. ARL, MSP and FGH generated the character matrix; MSP tested character correlations and assessed ancestral reconstruction. FGH provided access to museum collections, oversaw habitat/character coding, and helped to edit the manuscript. All authors read and approved the final manuscript.

## Authors’ information

Annie R Lindgren and Molly S Pankey are co-first authors.

## Supplementary Material

Additional file 1 **Supplementary Material: contents **[112-117]. Click here for file

Additional file 2 **Habitat and morphological character data used in analyses.** Abbreviations for Depth range coded from the literature are: “ukn” = data is not available for a particular species, “?” = a depth could not be defined as no species name was designated, “D” = depth in day, “N” = depth at night, “juv” = depth for juveniles, “OM” = ontogenetic migratory, “VM” = vertical migrator. Habitat states are 0 = pelagic, 1 = benthic/demersal. Character states are (0 = absent, 1 = present) for ANG, Branchial canal, Autogenic photophore, Bacteriogenic Photophore, and Right Oviduct. States for Cornea are: 0 = absent, 1 = 1-part cornea present, 2 = 2-part cornea present. Click here for file

Additional file 3 **Appendix 2.GenBank sequence identifiers (GI numbers) for loci used in analyses listed alphapbetically.** Changes to the species name used in this manuscript are listed in bold. Checkmarks denote taxa used in primary analysis (188-taxa), shown in Figure 1. Click here for file

Additional file 4 Appendix 3.GI numbers for all contaminant sequences. Click here for file
