# Supplementary material for: A multi-gene phylogeny of Cephalopoda supports convergent morphological evolution in association with multiple habitat shifts in the marine environment
Source: BMC Evol Biol. 2012 Jul 28;12:129. doi: 10.1186/1471-2148-12-129 (PMC3733422; doi:10.1186/1471-2148-12-129)
Supplement: Additional file 1 — Supplementary Material: contents [112-117]. [file 1471-2148-12-129-S1.doc]

# Supplementary Material: contents

ST1. Log-likelihood and BIC values for alternate partitioning strategies. p. 2

S1. Complete 408-taxon ML tree with bipartition supports from 1000 p. 3

nonparametric bootstraps. Every taxon listed in Appendix 1 was

included for analysis following methods outlined in main text.

S2**.** **Comparison of character-state model performance at key nodes**. p. 4

*Supplemental phylogenetic analyses: Methods and results* p. 5-8

*Figure legends for S3-16* p. 9

S3. Decapodiformes tree (unrooted) p. 10

S4. Quartet mapping p. 11

S5. Substitution saturation: 12S p. 13

S6. Substitution saturation: 16S p. 14

S7. Substitution saturation: 18S p. 15

S8. Substitution saturation: 28S p. 16

S9. Substitution saturation: cytochrome B p. 17

S10. Substitution saturation: cytochrome oxidase I p. 18

S11. Substitution saturation: histone 3a p. 19

S12. Substitution saturation: octopine dehydrogenase p. 20

S13. Substitution saturation: opsin p. 21

S14. Substitution saturation: pax6 p. 22

S15. RY-coded tree p. 23

S16. RY-coded Decapodiformes tree p. 24

**Supplementary Table 1. Log-likelihood and BIC values for alternate partitioning strategies.**

| **#** | **Partitioning strategy** | **Scheme** | | **LnL on 16-part tree** | **LnL alone** | **BIC** |
| --- | --- | --- | --- | --- | --- | --- |
|  |  |  | |  |  |  |
| ***1*** | **no partitioning** | ***12S + 16S + 18S + 28S + CO1 + CYTB + H3a + ODH +opsin + Pax6*** | | ***-198670*** | ***-198590*** | ***397366*** |
|  |  |  | |  |  |  |
| ***2*** | **mitochondrial**  **nuclear** | ***12S + 16S + CO1 + CYTB*** | | ***-194292*** | ***-194255*** | ***388636*** |
|  |  | ***18S + 28S + H3a + ODH +opsin + Pax6*** | |  |  |  |
|  |  |  | |  |  |  |
| ***2*** | **ribosomal**  **coding** | ***12S + 16S + 18S + 28S*** | | ***-197163*** | ***-197078*** | ***394378*** |
|  |  | ***CO1 + CYTB + H3a + ODH +opsin + Pax6*** | |  |  |  |
|  |  |  | |  |  |  |
| ***3*** | **ribosomal**  **coding: 1^st^+2^nd^ positions**  **coding: 3^rd^ positions** | ***12S + 16S + 18S + 28S*** | | ***-194794*** | ***-194717*** | ***389666*** |
|  |  | ***CO1 + CYTB + H3a + ODH +opsin + Pax6: 1^st^+2^nd^*** | |  |  |  |
|  |  | ***CO1 + CYTB + H3a + ODH +opsin + Pax6: 3^rd^*** | |  |  |  |
|  |  |  |  |  |  |  |
| ***4*** | **mt : ribosomal**  **nuclear: ribosomal**  **nuclear: coding**  **mt coding** | ***12S + 16S*** | ***18S + 28S*** | ***-191761*** | ***-191758*** | ***383627*** |
|  |  | ***CO1 + CYTB*** | ***H3a + ODH +opsin + Pax6*** |  |  |  |
|  |  |  |  |  |  |  |
| ***6*** | **mt : ribosomal**  **nuclear : ribosomal**  **nuclear: coding: 1^st^+2^nd^ positions**  **nuclear: coding: 3^rd^ positions**  **mt : coding: 1^st^+2^nd^ positions**  **mt : coding: 3^rd^ positions** | ***12S + 16S*** | ***18S + 28S*** | ***-188662*** | ***-188632*** | ***(best of 100:) 374498*** |
|  |  | ***CO1 + CYTB: 1^st^+2^nd^*** | ***H3a + ODH +opsin + Pax6: 1^st^+2^nd^*** |  |  |  |
|  |  | ***CO1 + CYTB: 3^rd^*** | ***H3a + ODH +opsin + Pax6: 3^rd^*** |  |  |  |
|  |  |  |  |  |  |  |
| ***8*** | **mt : ribosomal**  **nuclear : ribosomal**  **each coding gene** | ***12S + 16S*** | ***H3a*** | ***-190570*** | ***-190563*** | ***381349*** |
|  |  | ***18S + 28S*** | ***ODH*** |  |  |  |
|  |  | ***CO1*** | ***opsin*** |  |  |  |
|  |  | ***CYTB*** | ***Pax6*** |  |  |  |
|  |  |  |  |  |  |  |
| ***10*** | **each gene** | ***12S*** | ***CYTB*** | ***-190328*** | ***-190324*** | ***380916*** |
|  |  | ***16S*** | ***H3a*** |  |  |  |
|  |  | ***18S*** | ***ODH*** |  |  |  |
|  |  | ***28S*** | ***opsin*** |  |  |  |
|  |  | ***CO1*** | ***Pax6*** |  |  |  |
|  |  |  |  |  |  |  |
| ***16*** | **each ribosomal gene**  **each coding: 1^st^+2^nd^ positions**  **each coding: 3^rd^ positions** | ***12S*** | ***H3a: 1^st^+2^nd^*** | ***-187068*** | ***-187068*** | ***(best of 100:)***  ***377420*** |
|  |  | ***16S*** | ***H3a: 3^rd^*** |  |  |  |
|  |  | ***18S*** | ***ODH: 1^st^+2^nd^*** |  |  |  |
|  |  | ***28S*** | ***ODH: 3^rd^*** |  |  |  |
|  |  | ***CO1: 1^st^+2^nd^*** | ***opsin: 1^st^+2^nd^*** |  |  |  |
|  |  | ***CO1: 3^rd^*** | ***opsin: 3^rd^*** |  |  |  |
|  |  | ***CYTB: 1^st^+2^nd^*** | ***Pax6: 1^st^+2^nd^*** |  |  |  |
|  |  | ***CYTB: 3^rd^*** | ***Pax6: 3^rd^*** |  |  |  |

RAxML estimates substitution rates for data partitions independently. To provide appropriate partitions, we tested nine increasingly partitioned schemes on our optimal dataset. The 16-partition scheme yielded the best LnL score. BIC scores confirm this scheme yields significantly improved scores given the additional parameters.

**Figure S2**. **Comparison of character-state model performance at key nodes**. The distributions of likelihood score differences in the fit of opposing models (present or absent, for each character at each of 11 nodes) onto 1000 bootstrapped tress are shown. A character model that significantly outperforms the other yields a distribution that falls to the outside of the vertical bars. Distributions falling between the bars indicate that neither state substantially improves the fit of the character model to the trees.

Supplemental Methods

*Rooting effect on Decapodiformes*

Due to the substantial branch-length variation in cephalopods (Strugnell et al. 2005) and the continued spurious relationships among basal nodes in among benthic and pelagic basal squid lineages (Lindgren 2010), we further evaluated the effect that the inclusion Nautiloids and Octopodiformes had on decapodiform relationships.  Using the dataset constructed in the main text (cephalopods with four or more loci), we repeated the phylogenetic analyses including solely Decapodiformes taxa, under the same conditions and parameters as described in the main text.

*Likelihood quartet mapping*
 Using TreePuzzle (Schmidt et al 2002), we analyzed the amount of phylogenetic signal present within a dataset of decapodiform species in which at least four genes are present.  For 10000 iterations, quartet puzzling randomly samples one taxon from each of four specified groups (Sepiolids, Sepiids, Myopsids, Oegopsids) and infers the unrooted topology using a GTR model where substitution rates and gamma are estimated from the data. With our focus on understanding the relationships among the four major decapodiform lineages, *Idiosepius* and *Spirula* were omitted from the analysis.  Note that TreePuzzle does not permit data partitioning, as used in our RAxML analyses.

*Rate* *Heterogeneity*
 The breadth and scale of this dataset increases the potential effects of differing rates of evolution across both genes and lineages.  To determine the extent to which our dataset was subject to compositional heterogeneity, a partition homogeneity test on each gene within the complete dataset (409 taxonomic units) was conducted in PAUP, which uses a Χ^2^ distribution to evaluate the degree to which base frequencies for a sequence deviate from expected (Swofford 2002).

*Substitution saturation*

Because the Χ^2^ homogeneity test does not account for phylogenetic correlation of base frequencies, we employed an additional method to visualize potential substitution saturation similar to the techniques of Midnell and Honeycutt (1990) and DAMBE (Xia and Xie 2001).  For each pairwise taxon comparison, we calculated genetic distances under a GTR model (equal rates) at each gene in PAUP and plotted the observed proportion of transversion and transition substitutions for each gene, and for 1^st^ +2^nd^ sites and 3^rd^ sites in coding genes (uncorrected distances). The mean maximal genetic distance between key ordinal groups was estimated and plotted to serve as a reference:

Cephalopoda : Mean of distances between Nautiloids-Octopodids, Nautiloids-Oegopsids;

Octopodiformes : Mean of distances between *Vampyroteuthis* and any octopodid;

Decapodiformes : Mean of distances between Sepiolids-Oegopsids, and Sepiids-Oegopsids;

Oegopsida : Mean of distances between Ommastrephids-Gonatids, Gonatids-Histioteuthids, Ommastrephids-Enoploteuthids, Architeuthids-Enoploteuthids, Ommastrephids-Histioteuthids, Architeuthids-Ommastrephids, Histioteuthids-Cranchiids, Octopoteuthids-Cranchiids, Chiroteuthids-*Mastigoteuthis.*

Along with rooted and unrooted analyses of our unmodified codon-aligned dataset, we examined the effect of possible substitution saturation at the 3^rd^ positions on topology by creating modified datasets in which all 3^rd^ position sites of coding genes were coded as purines (adenine, guanine: R) or pyrimidines (cytosine, thymine: Y).  This coding strategy aims to minimize a potential source of bias in phylogenetic inference by allowing simply transitions and transversions to be considered at fast-evolving sites (Phillips et al. 2004). These modifications generated two additional datasets to be analyzed under the same parameters as described in the main text: one dataset of 188 cephalopod taxa contributing at least 4 genes for rooted analysis, and another with 135 decapodiforms contributing at least 4 genes for unrooted analysis.

Results

*Rooting effect on Decapodiformes*

When Nautiloids and Octopodiformes are excluded from the analysis, the resulting ML topology yields several differences (Figure S8). While interpreting bootstrap supports in an unrooted tree is not straightforward, since the true root could fall along any branch in theory, we note that *Spirula* and Sepiidae are recovered as adjacent groups in 66% of bootstrap replicates. Furthermore, although myopsids are sister to *Spirula*+Oegopsids+Bathyteuthoids in the rooted analysis, the myopsid group falls within the sepioid clan (Sepiida, Spirula, Idiosepiidae, Sepiolida lineages).in the unrooted analysis. The substantial topological rearrangement we observe suggests that our current outgroups, using currently available loci, may bias the branching order due to the extreme divergence that has occurred. Another possibility is that certain groups within the Decapodiformes, such as Idiosepiidae and Spirula, have experience elevated substitution rates relative to the remaining lineages. This alone or in combination with the effect of using very distant outgroups could account for the unconventional basal placement of Idiosepiidae in the main (rooted) analysis.

*Likelihood quartet mapping*
Likelihood quartet mapping of the decapodiform-only dataset in TreePuzzle indicated a slight preference (41.6% of quartets) for the topology ((Oegopsida, Myopsida),(Sepiolidae, Sepiidae)) (Figure S9). It should be noted however, that Teuthida and Uniductia are rooted hypotheses concerning the relationships among Decapodiformes, meaning that unrooted trees cannot be ascribed to support either hypothesis. This unrooted tree can be considered congruent with both hypotheses depending on root placement.  We note that the remaining two possible unrooted topologies combined received less than half as many quartets in the analysis (16.6% and 14.7% of quartets).

*Rate* *Heterogeneity*

Each gene within the complete dataset (409 species) was evaluated for base composition heterogeneity using the Χ^2^ homogeneity test in PAUP (Table ST2). The 3^rd^ codon-positions of mitochondrial coding genes *coI* and *cytb* deviate from expected base frequencies.  Likewise, the 3^rd^ position sites of *opsin* and *odh* also show considerable heterogeneity in base composition.  In fact, *opsin* appears to deviate from expected frequencies at each coding position, although the structure of the fragmentary nature of the opsin sequence alignment may artificially contribute to the apparent heterogeneity. While our estimates of compositional heterogeneity for coding genes is largely congruent with previous analyses (Strugnell at al. 2005; Strugnell and Nishiguchi 2007), we have additionally discovered that ribosomal genes 12S and 18S display severe compositional heterogeneity while 16S and 28S do not appear to deviate from expected frequencies.

**Table ST2. χ^2^ homogeneity test for compositional heterogeneity**

| *locus* | *df* | *χ2 across sites* | *χ2 on 1^st^ only* | *χ2 on 2^nd^ only* | *χ2 on 3^rd^ only* |
| --- | --- | --- | --- | --- | --- |
| *COI* | 741 | 776.1 | 335 | 103.2 | **2207****** |
| *CYTB* | 243 | **369.2****** | 79 | 87.34 | **896****** |
| *H3* | 270 | 89 | 11.84 | 4.3 | 258.6 |
| *OPSIN* | 252 | **606.7****** | **520.7****** | **348.4****** | **397.1****** |
| *PAX6* | 231 | 74.52 | 28.7 | 65.9 | 186.1 |
| *ODH* | 228 | 164.6 | 99.65 | 42.7 | **478.8****** |
| *12S* | 561 | **683.4****** |  |  |  |
| *16S* | 987 | 705.1 |  |  |  |
| *18S* | 309 | **421.8****** |  |  |  |
| *28S* | 357 | 110.7 |  |  |  |
| *combined* | 1224 | **37051**** |  |  |  |

*Substitution Saturation*

To further understand how well substitution rates reflect predicted genetic distances and how informative substitutions remain through deeper divergences, we plotted the observed proportion of transversion and transition substitutions for each gene (separately for 1^st^+2^nd^ sites and 3^rd^ sites in coding genes) against estimated genetic divergence under the GTR model (Figures S10-S19). When sequences reach saturation, any additional substitutions which may occur (Y-axis) fail to provide information as divergence time increases (X-axis) and a plateau in the data is observed (Mindell and Honeycutt 1990). Further, as transitions are expected to occur at a higher rate than transversions, transitions tend to saturate before transversions. This phenomena is evident in saturation plots as the two distributions appear to intersect as their respective slopes change.

Consistent with previous studies, we note that mitochondrial ribosomal genes 12S and 16S as well as the mitochondrial coding genes *coI* and *cytb* show elevated substitution rates compared with nuclear loci. Among these, 12S appears to suffer from worse saturation than 16S, although judging from the prevalence of points that fall beyond the mean genetic distance of Nautilus-Coleoids, presumably several lineages have experience especially high substitution rates in both genes. In the mitochondrial coding genes, we find evidence of extensive saturation at synonymous (3rd position codon) occurring much more recently than Decapodiformes ordinal divergences. However, *coI* (1+2positions) may be more robust to saturation at the divergence of Decapodiformes, while *cytb* appears to saturate quickly at all positions. We consider *cytb* poorly suited to resolve ordinal relationships within the Decapodiformes.

The increased scatter of the plots as genetic divergence increases presumably reflects the considerable substitution rate heterogeneity among lineages, as the scatter tends to be most extreme in sites identified in the ChiSq test as having heterogeneous composition. One exception to this trend is in nuclear ribosomal subunit 18S, which although significantly heterogeneous, appears to be the slowest evolving of the ribosomal genes considered here, and the least prone to saturation. Although nuclear 28S appears to saturate, the substitution rates of both transitions and transversions remain linear through the divergences within Decapodiformes, so we consider 28S a potentially suitable marker, along with 18S, for resolving ordinal relationships within Decapodiformes.  While synonymous sites (3^rd^ positions) always showed higher substitution rates compared with nonsynonomous sites (1^st^, 2^nd^ positions), nuclear coding genes (*opsin, histone3a, odh, pax6*) generally displayed little evidence of saturation prior to the major divergences within Octopodiformes and Decapodiformes. We also note that while overall rates of substitution are lower in nuclear coding genes than mitochondrial counterparts, the utility of nuclear loci to resolve ordinal relationships in the Decapodiformes appears more promising.

We anticipate that partitioning out the 3rd sites alleviates some of this noise, but if more genes (particularly nuclear and slow genes) can be included, then 3rd sites can be excluded or RYcoded without losing too much signal. Additional analyses examining the effect of both omitting 3rd positions entirely as well as RY coding all sites resulted in similar topologies as presented here with essentially no bootstrap support (data not shown)

We anticipate that many of the difficulties in resolving cephalopod relationships may be ameliorated by both the addition of many more suitable (i.e., less saturated, slow-evolving genes) and through the use of site-heterogeneous models (e.g., Lartillot et al. 2007)

*RY Coding at 3rd codon-positions*

Analysis of the RY-coded dataset produced many notable changes (Figure S20) including a reduction in bootstrap support for the placement of *V. infernalis*.   Also remarkable is the reshuffling of the branching orders within in the Decapodiformes: Idiosepiidae remains basal, but the subsequent branching suggests Sepiidae and Spirulidae are monophyletic and sister to Sepiolidae.  The Myopsids are recovered as sister to the sepioid-Spirulidae clade. However, bootstrap analysis fails to indicate any support for these relationships.  The monophyly of Bathyteuthoidea + Oegopsida remains well supported (100%).

However, when the Decapodiform-only dataset (135 taxa) was RY-coded, the inferred topology appears to be much more stable with respect to outgroup inclusion as no significant changes in topology occur; Sepiidae and Spirulidae form a monophyletic clade (63%) sister to Sepiolidae (Figure S21). While the support values recovered for either of these topologies do not offer any cause to prefer this result over the original dataset’s result, we emphasize that the effort to suppress the potential noise stemming from saturation at synonymous sites may be crucial in order to reduce long branch attraction as more data become available for cephalopod phylogeny.

Additional References

Lartillot, N., and H. Philippe. 2004. A Bayesian mixture model for across-site heterogeneities in the amino-acid replacement process. *Mol. Biol. Evol.* 21: 1095-1109.

Lindgren, A. R. 2010. Molecular inference of phylogenetic relationships among Decapodiformes (Mollusca: Cephalopoda) with special focus on the squid Order Oegopsida. *Mol. Phylogenet. Evol. 56*: 1-14.

Mindell, D.P., and R.L. Honeycutt. 1990. Ribosomal RNA in vertebrates: evolution and phylogenetic applications. Annu. Rev. Ecol. Evol. Syst. 21: 541-566.

Phillips, M.J., F. Delsuc, and D. Penny. 2004. Genome-scale phylogeny and the detection of systematic biases. *Mol. Biol. Evolution* 21:1455-1458.

Schmidt, H.A., K. Strimmer, M. Vingron, and A. von Haeseler. 2002. TREE-PUZZLE: maximum likelihood phylogenetic analysis using quartets and parallel computing. *Bioinformatics.* **18**:502-504.

Strugnell, J., and M.K. Nishiguchi. 2007. Molecular phylogeny of coleoid cephalopods (Mollusca : Cephalopoda) inferred from three mitochondrial and six nuclear loci: A comparison of alignment, implied alignment and analysis methods. *J. Molluscan Stud.* 73: 399-410.

Strugnell, J., M.D. Norman, J. Jackson, A.J. Drummond, and A. Cooper. 2005. Molecular phylogeny of coleoid cephalopods (Mollusca: Cephalopoda) using a multigene approach; the effect of data partitioning on resolving phylogenies in a Bayesian framework. *Mol. Phylogenet. Evol.* 37: 426-441.

Swofford, D. L. 2002. PAUP*. Phylogenetic Analysis Using Parsimony

(*and Other Methods). Version 4. Sinauer Associates, Sunderland,

Massachusetts.

Xia, X., and Z. Xie. 2001. DAMBE: Data analysis in molecular biology and evolution. J. Hered. 92: 371-373.

Supplemental Figure legends for S3-16:

Figure S3. Unrooted ML tree inferred in RAxML using only the data from the 135 decapodiform taxa shown in the ‘optimal’ 188-taxon cephalopod tree. Support values obtained from 1000 bootstrapped trees.

Figure S4. Likelihood Quartet Mapping analysis of phylogenetic signal within the decapodiform data. Idiosepiidae and Spirula were excluded from analysis. The results show that no single unrooted topology is supported after 10,000 unrooted trees are constructed randomly using one member of each group (‘quartet’). The likelihood with which a quartet is congruent with one of three topologies is reflected by its proximity to that topology’s corner. This analysis shows that 72.8% of quartets can be assigned in a non-ambiguous manner to a topology, but that there is signal conflict within the decapodiform data to support a particular topology.

S5-14. Saturation plots for the 10 loci used in this analysis. Genetic divergence of each pairwise sequence comparison, as estimated under a GTR model, is plotted along the X axis. The average number of either transitions (Ts) or transversions (Tv) calculated between each pair of sequences is plotted along the Y-axis. Vertical dashed lines represent estimates for major divergences (explained in supplemental methods). The extent to which these lines are spaced may potentially indicate how well a locus is suited to supply informative sites to resolve relationships at each divergence. Note that axes’ scales are not uniform.

Figure S15. ML phylogeny for optimal cephalopod dataset in which 3rd positions of coding sequence have been coded as simply purines or pyrimidines (RY-coded). Topology rooted with Nautilus. Supports from 1000 bootstraps have been enlarged at major nodes for clarity.

S16. Unrooted ML phylogeny for decapodiform taxa in which 3rd positions of coding sequence have been RY-coded. The same set of 135 decapodiform taxa as for Figure S8 were used. Bootstrap values from 1000 simulations are shown.
