# Supplementary material for: A multi-gene phylogeny of Cephalopoda supports convergent morphological evolution in association with multiple habitat shifts in the marine environment
Source: BMC Evol Biol. 2012 Jul 28;12:129. doi: 10.1186/1471-2148-12-129 (PMC3733422; doi:10.1186/1471-2148-12-129)
Supplement: Additional file 2 — Habitat and morphological character data used in analyses. Abbreviations for Depth range coded from the literature are: “ukn” = data is not available for a particular species, “?” = a depth could not be defined as no species name was designated, “D” = depth in day, “N” = depth at night, “juv” = depth for juveniles, “OM” = ontogenetic migratory, “VM” = vertical migrator. Habitat states are 0 = pelagic, 1 = benthic/demersal. Character states are (0 = absent, 1 = present) for ANG, Branchial canal, Autogenic photophore, Bacteriogenic Photophore, and Right Oviduct. States for Cornea are: 0 = absent, 1 = 1-part cornea present, 2 = 2-part cornea present. [file 1471-2148-12-129-S2.pdf]

Appendix 1. GenBank sequence identifiers (GI numbers) for loci used in analysis listed alphabetically. Changes to the species name used in this manuscript are listed in bold. Checkmarks denote taxa used in primary analysis (188–taxa), shown in Fig 1.

GenBank Name	Fig. 1	12S	16S	18S	28S	COI	cytb	H3	odh	opsin	pax
Abdopus abaculus		0	0	0	0	226088442	0	0	0	0	0
Abdopus aculeatus						226088444					
Abralia sp.		0	0	0	0	4003399	0	0	0	0	0
Abralia trigonura			498929								
Abralia veranyi	✔	0	209969975	209969995	209970067	209970147	0	209970192	0	0	0
Abraliopsis pacificus	✔	48994429	48994456							48994597	48994547
Abraliopsis pfefferi		0	0	49482097	49482150	0	0	50347033	0	0	0
Abraliopsis sp.			498928	34369157							
Abraliopsis sp. YJP 2002		0	0	0	34369182	0	0	0	0	0	0
Adelieledone piatkowski	✔	161611169	161611151						161611137	158828829	161611101
Adelieledone polymorpha	✔	129279685	18076692	0	0	15421837	161598182	0	45510967	45511068	45511020
Pareledone sp. AP 2001<br>**= Adelieledone sp. AP 2001**			18027468								
Afrololigo mercatoris		0	93004770	0	0	194474586	0	0	0	194474656	0
Alloteuthis africana			194474470			194474508				194474600	
Alloteuthis media		0	194474490	0	0	194474550	0	0	0	194474634	0
Alloteuthis subulata			194474505			194474580				194474650	
Alluroteuthis antarcticus		0	0	0	0	4927135	0	0	0	0	0
Ameloctopus litoralis							76359246				
Amphioctopus aegina		0	0	0	0	226088446	0	0	0	0	0
Octopus aegina<br>**= Amphioctopus aegina**		62005880	62005866				76359260				
Amphioctopus fangsiao		0	0	0	0	226088450	0	0	0	0	0
Octopus areolatus<br>**= Amphioctopus fangsiao**		62005878	18076177								
Amphioctopus kagoshimensis		0	0	0	0	226088456	0	0	0	0	0
Amphioctopus marginatus						207107966					
Octopus marginatus<br>**= Amphioctopus marginatus**		0	18076181	0	0	0	76359294	0	0	0	0
Amphioctopus cf neglectus<br>**= Amphioctopus neglectus**						207107970					
Amphioctopus cf ovulum<br>**= Amphioctopus ovulum**		0	0	0	0	207107972	0	0	0	0	0
Amphioctopus cf rex<br>**= Amphioctopus rex**						207107968					
Amphioctopus cf siamensis<br>**= Amphioctopus siamensis**		0	0	0	0	226088448	0	0	0	0	0
Amphitretus pelagicus						15421823					
Ancistrocheirus lesueuri		0	0	0	49482149	4003401	0	0	0	0	0
Ancistroteuthis lichtensteinii	✔		209969958	209970016	209970051	209970117		209970243			
Aphrodoctopus schultzei		0	18072925	0	0	0	0	0	0	0	0
Aphrodoctopus sp. JMS 2004		48994441	48994448								
Architeuthis dux	✔	2127256204	2127256203	49482087	82622108	2127256201	2127256202	38607259	0	0	0
Architeuthis sp.			498927		18072919	4003403					
Architeuthis sp. ARL 2008	✔	0	209969956	209969985	209970061	209970109	0	209970171	0	0	0
Architeuthis sp. JMS 2004	✔	48994443	48994455						48994503	48994593	

Appendix 1. GenBank sequence identifiers (GI numbers) for loci used in analysis listed alphabetically. Changes to the species name used in this manuscript are listed in bold. Checkmarks denote taxa used in primary analysis (188-taxa), shown in Fig 1.

GenBank Name	Fig. 1	12S	16S	18S	28S	COI	cytb	H3	odh	opsin	pax
Architeuthis sp. ML 2009		0	0	0	0	255642897	0	0	0	0	0
Argonauta argo		62005877	62005863			62084161					
Argonauta nodosa	✔	45510911	45510935	49482067	0	4003405	76359244	50346987	45510951	45511050	0
Asperoteuthis nesisi	✔	167538900	167538899	209970022	209970068	167538892					
Bathypolypus arcticus		0	82622208	49482070	0	4003407	0	0	0	0	0
Bathypolypus sp.			16944397								
Bathypolypus sp. JMS 2004	✔	48994418	48994446	0	0	0	0	0	48994469	48994571	48994525
Bathypolypus sponsalis		116829864	18072838				239735781				
Bathypolypus valdiviae	✔	48994417	0	0	0	15421831	0	0	48994467	0	48994523
Bathyteuthis abyssicola	✔	48994432	209969915	49482088	49482142	4003409		50347019	48994493	48994603	48994553
Bathyteuthis berryi	✔	48762894	48762909	0	0	48762876	0	0	48994318	48994356	48994386
Bathyteuthis sp. A	✔		209969938	209969988	209970031	209970077		209970177			
Batoteuthis skolops	✔	0	209969916	49482089	49482143	0	0	50347021	0	0	0
Bentheledone sp. CYV 2001						15421843					
Bentheledone sp. HBH		0	18072926	0	0	0	0	0	0	0	0
Benthoctopus eureka							239735783				
Benthoctopus eureka 1 **= Benthoctopus eureka**		116829865	116829855	0	0	0	0	0	0	116829806	0
Benthoctopus johnsonianus	✔	116829867	116829858				239735785			116829812	
Benthoctopus cf levis **= Benthoctopus levis**		0	116829857	0	0	0	0	0	0	116829810	0
Benthoctopus levis		226350502									
Benthoctopus normani		0	0	0	0	0	239735803	0	0	116829814	0
Benthoctopus normani 3 **= Benthoctopus normani**		116829868	116829859								
Benthoctopus oregonensis		239735813	239735811	0	0	0	239735787	0	0	0	0
Benthoctopus cf profundorum **= Benthoctopus profundorum**		239735816	239735810				239735797				
Benthoctopus rigbyae		226350505	226350509	0	0	0	239735805	0	0	0	0
Benthoctopus sp.			18072839								
Benthoctopus sp. A		239735814	239735808	0	0	0	239735791	0	0	0	0
Benthoctopus sp. A JMS 2004									45510963	45511064	45511016
Benthoctopus sp. B		239735820	0	0	0	0	239735801	0	0	0	0
Benthoctopus sp. CYV 2001						15421833					
Benthoctopus sp. JMS 2004	✔	48994419	0	0	0	0	0	0	48994471	48994573	48994527
Benthoctopus sp. MV		239735821									
Benthoctopus thielei		226350504	226350508	0	0	0	0	0	0	0	0
Benthoctopus yaquinae	✔	239735818	239735807	34369159			239735789				
Berryteuthis anonychus	✔	58202079	209969954	209970002	209970048	209970105	0	209970201	0	0	0
Berryteuthis magister	✔	62005902	209969979	209970026	209970070	58202168		209970203			
Bolitaena pygmaea	✔	45510925	0	0	0	4003425	0	0	45510977	45511078	45511030
Brachioteuthis beanii						4003411					
Brachioteuthis sp. 1		0	209969917	0	0	0	0	0	0	0	0
Brachioteuthis sp. 2	✔		209969939	209969989	209970032	209970079		209970179			
Brachioteuthis sp. 3	✔	0	209969940	209969990	209970033	209970081	0	209970181	0	0	0
Brachioteuthis sp. ARL 2004				49482090	49482144			50347023			

Appendix 1. GenBank sequence identifiers (GI numbers) for loci used in analysis listed alphabetically. Changes to the species name used in this manuscript are listed in bold. Checkmarks denote taxa used in primary analysis (188-taxa), shown in Fig 1.

GenBank Name	Fig. 1	12S	16S	18S	28S	COI	cytb	H3	odh	opsin	pax
Callistoctopus aspilosomatis		0	0	0	0	226088466	0	0	0	0	0
Octopus aspilosomatis<br>**= Callistoctopus aspilosomatis**			18076683				76359264				
Callistoctopus luteus		0	0	0	0	207107974	0	0	0	0	0
Octopus macropus<br>**= Callistoctopus macropus**			83306180								
Callistoctopus minor		0	0	0	0	226088496	0	0	0	0	0
Callistoctopus ornatus						226088472					
Octopus ornatus<br>**= Callistoctopus ornatus**	✔	62005883	18076183	0	0	0	0	0	48994354	48994384	48994414
Callistoctopus sp. NSMTMo74815						207107980					
Chiroteuthis calyx	✔	0	209969953	209969999	209970047	209970103	0	209970211	0	0	0
Chiroteuthis mega	✔		209969941	209969998	209970034	209970083		209970215			
Chiroteuthis veranyi	✔	0	209969962	209970000	209970054	209970125	0	209970213	0	0	0
Chtenopteryx sicula	✔	34542039	34542069	209969986	209970043	209970097		209970173			
Chtenopteryx sp. ARL 2008	✔	0	209969967	209969987	209970060	209970135	0	209970175	0	0	0
Cirroctopus glacialis			28629084								
Grimpoteuthis glacialis<br>**= Cirroctopus glacialis**		0	0	0	0	15421817	0	0	0	0	0
Cirroctopus hochbergi			21912808								
Cirroteuthis muelleri		0	28629064	0	0	0	0	0	0	0	0
Cirrothauma murrayi			28629062	49482061		4003417					
Cistopus cf indicus<br>**= Cistopus indicus**		0	0	0	0	207107982	0	0	0	0	0
Cistopus indicus			18073263				76359248				
Cistopus sp. JMS 2004		48994421	0	0	0	0	0	0	48994475	48994577	48994531
Cranchia scabra	✔	48994436	209969918	49482092	49482145	4003419		50347025	48994519	48994611	48994561
Cycloteuthis sirventyi	✔	0	209969920	49482094	49482147	4003421	0	50347029	0	0	0
Discoteuthis discus	✔		209969945	209969994	209970039	209970091		209970227			
Discoteuthis laciniosa	✔	0	209969921	49482095	49482148	4003423	0	50347031	0	0	0
Doratosepion sp. LB 2005		77157303									
Loligo gahi<br>**= Doryteuthis gahi**	✔	0	93004761	0	0	5678658	0	0	0	0	0
Loligo opalescens<br>**= Doryteuthis opalescens**	✔	2402666124	2402666123			2402666121	2402666122		12055096		1778016
Loligo pealei<br>**= Doryteuthis pealeii**	✔	0	56567271	34369176	34369209	18026439	0	38607257	0	0	0
Loligo plei<br>**= Doryteuthis plei**	✔		93004765			14120087					
Dosidicus gigas	✔	1548161314	1548161313	209970009	209970049	1548161311	1548161312	209970229	0	0	0
Eledone cirrhosa	✔	48994420	18073265	49482072					48994473	48994575	48994529
Eledone massyae		0	18073266	0	0	0	0	0	0	0	0
Eledone moschata			18073267								
Enoploteuthis galaxias		0	3175997	0	0	0	0	0	0	0	0
Enoploteuthis higginsi	✔	48994430	3175998						48994495	48994599	48994549
Enoploteuthis leptura	✔	0	209969922	49482098	49482151	0	0	50347035	0	0	0

Appendix 1. GenBank sequence identifiers (GI numbers) for loci used in analysis listed alphabetically. Changes to the species name used in this manuscript are listed in bold. Checkmarks denote taxa used in primary analysis (188-taxa), shown in Fig 1.

GenBank Name	Fig. 1	12S	16S	18S	28S	COI	cytb	H3	odh	opsin	pax
Enoploteuthis reticulata			498933			4003427					
Enoploteuthis sp. BL 2001		0	0	0	18375783	0	0	0	0	0	0
Enteroctopus dofleini	✔	62005876	45510940			62084159	239735795		45510965	45510966	45511018
Octopus magnificus		0	18076180	0	0	0	0	0	0	0	0
**= Enteroctopus magnificus**											
Eucleoteuthis luminosa	✔		209969960	209970010		209970119		209970225			
Euprymna berryi	✔	34542045	14161379	0	34542098	13195587	0	0	0	0	0
Euprymna hyllebergi	✔	34542048	34542074		34542099	34542132					
Euprymna morsei		62005906	34542070	0	34542100	0	0	0	0	0	0
Euprymna scolopes	✔	34542046	34542072		34542097	34542128			48994346	48994378	21667880
Euprymna sp.		0	498935	0	0	0	0	0	0	0	0
Euprymna tasmanica	✔	34542047	34542073		34542108	34542130				48994587	48994541
Galiteuthis armata	✔	0	0	209970023	209970053	209970151	0	209970188	0	0	0
Galiteuthis sp. JMS 2004	✔	48994433	48994461						48994483	48994605	48994555
Gonatopsis borealis		58202061	58202086	0	0	4927137	0	0	0	0	0
Gonatopsis japonicus		58202058	58202080			58202112					
Gonatopsis octopedatus	✔	58202060	58202085	209970027	209970071	58202122	0	0	0	0	0
Gonatopsis cf okutanii			209969981	209970028		209970161					
**= Gonatopsis okutanii**											
Gonatopsis sp. 01		58202066	58202092	0	0	58202136	0	0	0	0	0
Gonatopsis sp. ARL 2008	✔		209969951	209970003	209970045			209970199			
Gonatus antarcticus	✔	0	58202093	49482102	49482155	58202138	0	50347043	0	0	0
Gonatus berryi			58202095			4003429					
Gonatus californiensis		0	0	0	0	5052409	0	0	0	0	0
Gonatus fabricii	✔	58202067	58202094	49482103	49482156	4927139		50347045			
Gonatus kamtschaticus		58202071	58202099	0	0	58202148	0	0	0	0	0
Gonatus madokai		58202075	58202106			58202162					
Gonatus cf onyx		58202074	58202104	0	0	0	0	0	0	0	0
Gonatus onyx						4003431					
Gonatus pyros		0	58202105	0	0	58202160	0	0	0	0	0
Gonatus tinro		58202068	58202097			58202142					
Graneledone antarctica	✔	161611174	161611156	0	0	15421839	0	0	0	158828843	161611135
Graneledone pacifica				34369169							
**= Graneledone borealopacifica**											
Graneledone boreopacifica	✔	161611173	161611155	0	0	15421841	0	0	161611149	158828839	161611131
Graneledone verrucosa	✔	45510922	82561543	49482073		4003433		50346991	45510973	158828841	45511024
Grimalditeuthis bonplandi	✔	0	209969942	209970001	209970035	209970085	0	209970217	0	0	0
Grimpella thaumastocheir							76359250				
Grimpoteuthis sp.		0	93004785	0	0	5678678	0	0	0	0	0
Grimpoteuthis sp. Challenger			28629089								
Grimpoteuthis sp. CYV 2001		0	0	0	0	15421819	0	0	0	0	0
Grimpoteuthis sp. Discovery			28629088								
Haliphron atlanticus	✔	48994416	48994445	49482065	0	15421821	0	0	48994352	48994569	48994521
Haliphron sp. ARL 2004								50346986			
**= Haliphron atlanticus**											
Hapalochlaena fasciata		0	0	0	0	226088474	76359252	0	0	0	0

Appendix 1. GenBank sequence identifiers (GI numbers) for loci used in analysis listed alphabetically. Changes to the species name used in this manuscript are listed in bold. Checkmarks denote taxa used in primary analysis (188-taxa), shown in Fig 1.

GenBank Name	Fig. 1	12S	16S	18S	28S	COI	cytb	H3	odh	opsin	pax
Hapalochlaena lunulata		62005882	18073264			226088476					
Hapalochlaena maculosa	✔	45510916	45510938	0	0	226088478	76359256	0	45510959	45511060	45511012
Hapalochlaena sp. 1 MG 2004							76359254				
Helicocranchia pfefferi		0	93004784	0	0	5678671	0	0	0	0	0
Heterololigo bleekeri	✔	97906254	97906253			979062511	97906252				
Heteroteuthis hawaiiensis	✔	34542063	34542089	49482077	82622107	34542160	0	50346997	48994344	48994376	48994406
Histioteuthis bonellii	✔		209969964	209970005	209970056	209970129		209970209			
Histioteuthis corona	✔	0	209969927	49482104	49482157	0	0	50347047	0	0	0
Histioteuthis hoylei	✔		209969928	49482105	49482158	4003439		50347049			
Histioteuthis miranda	✔	0	209969971	209970006	209970064	209970141	0	209970207	0	0	0
Histioteuthis oceani	✔	48994428	48994460						48994487	48994595	48994545
Histioteuthis reversa	✔	0	209969947	49482106	209970041	209970143	0	209970205	0	0	0
Histioteuthis sp.			498936								
Histioteuthis sp. YJP 2002		0	0	34369171	0	0	0	0	0	0	0
Idiosepius biserialis		157932131	157863593								
Idiosepius macrocheir		157932166	157863628	0	0	0	0	0	0	0	0
Idiosepius notoides	✔	157932168	3618170						45510979	45511080	45511032
Idiosepius paradoxus		62005903	62005892	0	0	62084191	0	0	0	0	0
Idiosepius picteti		157932192	157863661								
Idiosepius pygmaeus	✔	34542043	209969911	49482082	0	4003441	0	50347007	0	0	0
Illex argentinus			498937			87042902					
Illex coindetii	✔	48994437	209969934	49482114	49482167	48762892	0	50347065	48994505	48994613	48994563
Illex cf illecebrosus<br>= **Illex illecebrosus**			18253970								
Illex illecebrosus		0	0	0	0	87042834	0	0	0	0	0
Japatella heathi<br>= **Japetella diaphana**						15421825					
Japetella diaphana	✔	45510924	18073269	49482068	0	0	0	0	45510975	45511076	45511028
Joubiniteuthis portieri	✔	165868244	165868255	49482107	49482160	4003445		50347053			
Joubiniteuthis sp. JMS 2004		0	0	0	0	0	0	0	48994326	48994360	48994390
Kondakovia sp. ARL 2008	✔		209969983	209970030		209970165		209970247			
Leachia atlantica	✔	0	209969919	49482093	49482146	0	0	50347027	0	0	0
Leachia lemur	✔		209969968	209969991	209970063	209970137		209970186			
Leachia pacifica		0	496355	0	0	0	0	0	0	0	0
Lepidoteuthis grimaldii	✔		209969930	49482108	49482161	4003447		50347055			
Liocranchia valdiviae		0	498940	0	0	4003449	0	0	0	0	0
Loligo forbesi	✔	45510930	498938			5678661			45510987	45511086	45511040
Loligo reynaudii		0	93004766	0	0	5678665	0	0	0	0	0
Loligo sp. AL9407 Loligo 1K			18253969								
Loligo vulgaris	✔	77157317	498939	0	0	5678656	0	0	13561066	0	0
Loliolus japonica			93004768			5678666	145244494				
Loliolus sp. JMS 2004	✔	48762902	48762913	0	0	48762884	0	0	48994340	48994372	48994402
Lolliguncula brevis		48762898	93004769			5678655			48994332	48994364	48994394
Lolliguncula diomedeae	✔	0	209969959	209969984	209970062	209970073	0	209970169	0	0	0
Luteuthis dentatus			21912935								
Lycoteuthis lorigera	✔	0	209969973	209970020	209970065	209970145	0	209970198	0	0	0

Appendix 1. GenBank sequence identifiers (GI numbers) for loci used in analysis listed alphabetically. Changes to the species name used in this manuscript are listed in bold. Checkmarks denote taxa used in primary analysis (188-taxa), shown in Fig 1.

GenBank Name	Fig. 1	12S	16S	18S	28S	COI	cytb	H3	odh	opsin	pax
Octopus maorum			18076684				76359292				
**= Macroctopus maorum**											
Magnapinna sp. ARL 2008	✔	0	209969943	209970018	209970036	209970087	0	209970237	0	0	0
Mastigoteuthis agassizii	✔	165868248	165868260	49482109	49482162	169930488		50347057			
Mastigoteuthis latipinna		0	0	0	0	5678673	0	0	0	0	0
**= Mastigoteuthis cordiformis**											
Mastigoteuthis hjorti	✔	165868251	165868261	209970007	209970037	209970089		209970221			
Mastigoteuthis magna	✔	165868246	165868258	49482110	49482163	4003455	0	50347059	0	0	0
Mastigoteuthis microlucens		165868241	165868252			169930486					
Mastigoteuthis sp. M1		165868245	165868256	0	0	169930494	0	0	0	0	0
Megaleledone senoi						15421847					
**= Megaleledone setebos**											
Megaleledone setebos	✔	129279686	129279664	0	0	0	0	0	161611139	154813166	161611129
Megalocranchia fisheri	✔	48994434							48994491	48994607	48994557
Megalocranchia sp. ARL 2008		0	209969944	209969993	209970038	209970123	0	209970183	0	0	0
Mesonychoteuthis hamiltoni	✔		209969977	209970024		209970153		209970190			
Metasepia pfefferi		77157304	0	0	0	0	0	0	0	0	0
Metasepia tullbergi	✔	62241210	62241202			62241222	242117741		48994350	48994382	48994412
Onykia knipovitchi		0	209969936	49482117	49482170	4927143	0	50347071	0	0	0
**= Moroteuthis knipovitchi**	✔										
Nautilus belauensis			571356								
Nautilus macromphalus	✔	944906654	9449066533	17385427	0	911773921	944906652	0	0	0	0
Nautilus pompilius	✔	48994439	38607021	34369177		18026437					48994567
Nautilus repertus		0	571355	0	0	0	0	0	0	0	0
Nautilus scrobiculatus			571333	18026322							
Nautilus stenomphalus		0	571332	0	0	0	0	0	0	0	0
Neorossia caroli			117960047								
Neoteuthis thielei	✔	0	209969931	49482111	49482164	0	0	50347061	0	0	0
Notonykia sp. ARL 2008	✔		209969948	209970014	209970042	209970115		209970245			
Nototodarus gouldi		0	38641271	0	0	0	0	0	0	0	0
Nototodarus hawaiiensis			498942								
Nototodarus sp. GD 2007		0	148337316	0	0	0	0	0	0	0	0
Octopoteuthis megaptera	✔		209969974	209970008	209970066	209970075		209970235			
Octopoteuthis nielseni	✔	48994431	48994457	49482112	49482165	4003459	0	209970231	48994515	48994601	48994551
Octopoteuthis sicula	✔		209969933	49482113	49482166			50347063			
Octopus aculeatus		0	0	0	0	0	76359258	0	0	0	0
Octopus alpheus							76359262				
Octopus australis		0	0	0	0	0	76359266	0	0	0	0
Octopus berrima	✔	45510913	45510936				76359268		45510953	45511054	45511006
Octopus bimaculoides	✔	45510917	18076178	0	0	15421827	0	0	45510961	45511062	45511014
Octopus bocki			18076686								
Octopus bunurong		0	0	0	0	0	76359270	0	0	0	0
Octopus californicus			16944718			15421829					
Octopus conispadiceus		62005885	62005871	0	0	226088482	0	0	0	0	0
Octopus cyanea	✔	62005884	18076207			226088484	76359272				
Octopus dierythraeus		0	0	0	0	0	76359276	0	0	0	0

Appendix 1. GenBank sequence identifiers (GI numbers) for loci used in analysis listed alphabetically. Changes to the species name used in this manuscript are listed in bold. Checkmarks denote taxa used in primary analysis (188-taxa), shown in Fig 1.

GenBank Name	Fig. 1	12S	16S	18S	28S	COI	cytb	H3	odh	opsin	pax
Octopus exannulatus							76359278				
Octopus graptus		0	0	0	0	0	76359280	0	0	0	0
Octopus hongkongensis		186704370	186704369			226088490					
Octopus incella		0	0	0	0	226088500	0	0	0	0	0
Octopus joubini						38607157		38607255			
Octopus kagoshimensis		0	18076682	0	0	0	76359284	0	0	0	0
Octopus kaurna	✔	45510914	45510937						45510955	45511056	45511008
Octopus laqueus		186704374	186704373	0	0	226088502	0	0	0	0	0
Octopus longispadiceus						226088494					
Octopus luteus		0	18076179	0	0	0	0	0	0	0	0
Octopus maya			226424604								
Octopus mimus		0	16944714	0	0	0	0	0	0	0	0
Octopus minor		62005879	62005865			62084165					
Octopus mototi		0	18076182	0	0	0	76359296	0	0	0	0
Octopus ocellate sp							76359298				
Octopus ocellatus	✔	893374134	893374133	0	0	893374131	893374132	0	0	0	0
Octopus oculifer							76359300				
Octopus oliveri		0	0	0	0	226088480	0	0	0	0	0
Octopus pallidus			18076184				76359302				
Octopus parvus		62005875	62005861	0	0	226088504	0	0	0	0	0
Octopus rubescens	✔	45510915	18076185						45510957	45511058	45511010
Octopus salutii		0	16944719	0	0	0	0	0	0	0	0
Octopus sasakii		62005881	62005867			62084169					
Octopus sp.		0	18076186	0	0	0	0	0	0	0	0
Octopus sp. 10 MG 2004							76359288				
Octopus sp. 5 MG 2004		0	0	0	0	0	76359282	0	0	0	0
Octopus sp. 8 MG 2004							76359286				
Octopus sp. hakutoensis		186704366	186704365	0	0	0	0	0	0	0	0
Octopus sp. HBH 6			18076687								
Octopus sp. NSMT Mo75218		0	0	0	0	207107984	0	0	0	0	0
Octopus sp. OM853		62086208	62086207			62086209					
Octopus sp. OM870		62086204	62086203	0	0	62086205	0	0	0	0	0
Octopus sp. OM949		62086212	62086211			62086213					
Octopus sp. TL 2006		0	118430808	0	0	0	0	0	0	0	0
Octopus sp. xSA MG 2004							76359290				
Octopus tehuelchus		0	18076191	0	0	0	0	0	0	0	0
Octopus tetricus						4003461	76359304				
Octopus variabilis		0	161016099	0	0	0	0	0	0	0	0
Octopus vulgaris	✔	537938874	537938873			537938871	537938872			116829804	
Octopus wolfi		0	18076685	0	0	226088506	0	0	0	0	0
Ocythoe tuberculata				49482069							
Ommastrephes bartramii	✔	62005898	209969935	49482115	49482168	4003463	0	50347067	48994330	48994362	48994392
Onychoteuthis banksii						154347379					
Onychoteuthis cf banksii = **Onychoteuthis banksii**	✔	0	209969966	209970012	209970058	0	0	209970249	0	0	0
Onychoteuthis borealijaponica						154347377					

Appendix 1. GenBank sequence identifiers (GI numbers) for loci used in analysis listed alphabetically. Changes to the species name used in this manuscript are listed in bold. Checkmarks denote taxa used in primary analysis (188-taxa), shown in Fig 1.

GenBank Name	Fig. 1	12S	16S	18S	28S	COI	cytb	H3	odh	opsin	pax
Onychoteuthis compacta		0	3176101	0	0	4003465	0	0	0	0	0
Onychoteuthis sp.			3176102								
Onychoteuthis sp. B3 JMS 200	✔	48994442	48994454	0	0	0	0	0	48994485	48994591	0
Onychoteuthis sp. BC			3176100								
Onykia carriboea	✔	0	209969950	209970015	209970044	209970099	0	209970239	0	0	0
Onykia ingens			498941			154347375					
Onykia lonnbergii		0	0	0	0	154347373	0	0	0	0	0
Onykia robsoni			3176080			154347371					
Onykia robusta	✔	0	3176079	209970013	209970050	154347369	0	209970241	0	0	0
Onykia sp			3176096								
Onykia sp. Moroteuthis		0	0	0	18076659	0	0	0	0	0	0
Onykia sp. A						154347381					
Opisthoteuthis californiana		0	21912932	0	0	0	0	0	0	0	0
Opisthoteuthis sp. ARL 2004 **= Opisthoteuthis californiana**								50346980			
Opisthoteuthis depressa		62005886	62005872	0	0	62084179	0	0	0	0	0
Opisthoteuthis hardyi			28629082								
Opisthoteuthis massyae	✔	45510910	21912931	0	0	0	0	0	45510949	45511048	45511000
Opisthoteuthis sp			18076193								
Opisthoteuthis sp. B PCHH2001		0	21912933	0	0	0	0	0	0	0	0
Opisthoteuthis sp. CYV 2001						15421815					
Opisthoteuthis sp. JMS 2004		48994440	48994444	0	0	0	0	0	0	0	0
Opisthoteuthis sp. Thallassa			28629083								
Ornithoteuthis antillarum	✔	0	209969923	49482099	49482152	0	0	50347037	0	0	0
Pareledone aequipapillae	✔	129279691	129279669						129279547	154813169	161611107
Pareledone albimaculata	✔	129279693	129279671	0	0	0	0	0	129279551	154813170	161611109
Pareledone aurata	✔	129279689	129279667						129279543	154813168	161611103
Pareledone charcoti	✔	129279687	129279665	0	0	15421835	0	0	129279541	154813167	161611105
Pareledone cornuta	✔	129279697	129279675						129279559	154813171	161611111
Pareledone felix	✔	129279695	129279673	0	0	0	0	0	129279557	129279528	0
Pareledone panchroma	✔	129279705	129279683						129279575	154813175	161611115
Pareledone serperastrata	✔	129279699	129279677	0	0	0	0	0	129279563	154813172	161611113
Pareledone subtilis	✔	129279701	129279679						129279567	154813173	161611117
Pareledone turqueti	✔	129279703	129279681	0	0	0	0	0	45510969	45511070	45511022
Pholidoteuthis adami	✔		209969970	209970021		209970139		209970253			
Planctoteuthis danae		0	0	0	0	5678669	0	0	0	0	0
Planctoteuthis levimana	✔		209969963	209969997	209970055	209970127		209970219			
Psychroteuthis glacialis		0	0	0	0	4927145	0	0	0	0	0
Psychroteuthis sp. ARL 2004 **= Psychroteuthis glacialis**	✔		209969937	49482118	49482171			50347073			
Pterygioteuthis gemmata	✔	0	209969924	49482100	49482153	0	0	50347039	0	0	0
Pterygioteuthis giardi hoylei	✔		209969978	209970025	209970069	209970155		209970196			
Pterygioteuthis microlampas	✔	48762895	209969969	209969996	209970059	209970133	0	209970194	48994322	48994358	48994388
Pyroteuthis addolux						4003469					
Pyroteuthis margaritifera		0	0	49482101	49482154	0	0	50347041	0	0	0
Rondeletiola minor	✔	34542060	34542086		34542095	34542154					

Appendix 1. GenBank sequence identifiers (GI numbers) for loci used in analysis listed alphabetically. Changes to the species name used in this manuscript are listed in bold. Checkmarks denote taxa used in primary analysis (188-taxa), shown in Fig 1.

GenBank Name	Fig. 1	12S	16S	18S	28S	COI	cytb	H3	odh	opsin	pax
Rossia bipapillata		34542059	34542085	0	0	0	0	0	0	0	0
Rossia macrosoma			498944								48994543
Rossia pacifica	✔	62005904	62005893	0	0	5353806	0	0	48994342	48994374	48994404
Rossia palpebrosa	✔		209969908	49482078	49482135	4003471		50346999			
Rossia sp.		0	93004781	0	0	5678677	0	0	0	0	0
Scaeurgus unicirrhus			16944720								
Selenoteuthis scintillans	✔	0	209969946	209970019	209970040	209970093	0	0	0	0	0
Semirossia tenera		38374163	38374164			38374165					
Sepia aculeata		77157322	14161382	0	0	13195589	0	0	0	0	0
Sepia andreana						242117663	242117693				
Sepia apama	✔	48994424	48994451	0	0	0	0	0	0	48994583	48994537
Sepia aureomaculata						242117665	242117695				
Sepia bertheloti		0	34559533	0	0	242117667	242117697	0	0	0	0
Sepia elegans	✔	34542042	34542066		18026376	242117669	242117701	38607261			
Sepia elliptica		77157305	77157331	0	0	0	0	0	0	0	0
Sepia esculenta	✔	1531239214	1531239213			1531239211	1531239212				
Sepia filibrachia		77157318	0	0	0	0	0	0	0	0	0
Sepia furcata						46947386					
Sepia gibba		0	0	0	0	242117671	242117703	0	0	0	0
Sepia hierredda			34559531			34996415					
Sepia hirunda		0	0	0	0	46947384	0	0	0	0	0
Sepia kobiensis	✔	62241208	62241200			62909952	242117705				
Sepia latimanus	✔	62241207	62241199	0	0	62241218	242117707	0	0	0	0
Sepia lorigera	✔	62909941	62909937			62909946	242117711				
Sepia lycidas	✔	62241206	62241198	0	0	62241216	242117713	0	0	0	0
Sepia madokai			77157330			242117675	242117715				
Sepia officinalis	✔	892552844	892552843	49482076	49482134	892552841	892552842	50346995	13561068	0	121495688
Sepia opipara		77157308				4003475					
Sepia orbignyana		77157309	498950	0	0	0	0	0	0	0	0
Sepia papuensis		77157310	498951								
Sepia pardex	✔	62909940	62909936	0	0	62909944	242117717	0	0	0	0
Sepia peterseni	✔	62241209	62241201			62241220	242117719				
Sepia pharaonis	✔	77157311	116041621	0	0	14028758	242117721	0	48994348	48994380	48994410
Sepia plangon		77157312									
Sepia prashadi		0	0	0	0	242117679	242117723	0	0	0	0
Sepia recurvirostra	✔	77157321	77157329			242117681	242117725				
Sepia rex		77157314	0	0	0	0	0	0	0	0	0
Sepia robsoni			14150654			13195591					
Sepia smithi		77157313	77157333	0	0	0	0	0	0	0	0
Sepia sp. SI0604	✔	62909942	62909938			62909948	242117733				
Sepia subtenuipes		0	0	0	0	0	242117735	0	0	0	0
Sepia tenuipes	✔					242117683	242117727				
Sepia tokioensis	✔	0	0	0	0	242117685	242117729	0	0	0	0
Sepia whitleyana		77157315									
Sepiadarium austrinum	✔	48994423	48994450	0	0	0	0	0	48994479	48994581	48994535
Sepiadarium kochi	✔	62005907	34542087		34542111	38154317					

Appendix 1. GenBank sequence identifiers (GI numbers) for loci used in analysis listed alphabetically. Changes to the species name used in this manuscript are listed in bold. Checkmarks denote taxa used in primary analysis (188-taxa), shown in Fig 1.

GenBank Name	Fig. 1	12S	16S	18S	28S	COI	cytb	H3	odh	opsin	pax
Sepiella inermis	✔	0	209969906	49482075	49482133	0	0	209970167	0	0	0
Sepiella japonica	✔	77157302	48994452			242117691	242117737		48994481	48994585	48994539
Sepiella maindroni		62241211	62241203	0	0	13195581	0	0	0	0	0
Sepietta neglecta	✔	34542056	34542082		34542105	34542148					
Sepietta obscura	✔	34542057	34542083	0	34542104	34542150	0	0	0	0	0
Sepietta oweniana		34542058	34542084			34542152					
Sepietta sp.		0	498954	0	0	0	0	0	0	0	0
Sepiola affinis	✔	34542050	209969909	49482079	34542106	34542136		50347001			
Sepiola atlantica	✔	34542055	34542081	0	34542102	34542146	0	0	0	0	0
Sepiola birostrata	✔	34542049	34542075		34542103	34542134					
Sepiola intermedia	✔	34542052	34542078	0	34542109	34542140	0	0	0	0	0
Sepiola ligulata	✔	34542051	34542077		34542101	34542138					
Sepiola robusta	✔	34542053	34542079	0	34542110	34542142	0	0	0	0	0
Sepiola rondeleti		34542054	34542080			34542144					
Sepiola sp		0	498952	0	0	0	0	0	0	0	0
Sepiolina nipponensis	✔	34542062	34542088		34542091	34542158					
Sepioloidea lineolata	✔	48994422	48994449	0	0	4003477	0	0	48994477	48994579	48994533
Sepioteuthis australis	✔	48762899	93004772			4003479			48994334	48994366	48994396
Sepioteuthis lessoniana	✔	892554074	892554073	49482085	49482140	890008521	892554072	50347013	48994336	48994368	48994398
Sepioteuthis sepioidea			93004775			5678651					
Spirula spirula	✔	34542040	498953	49482081	18076661	34542122	0	50347005	45510983	45511084	45511036
Stauroteuthis gilchristi	✔	45510909	45510933						45510947	45511046	45510998
Stauroteuthis sp. Bahia		0	28629076	0	0	0	0	0	0	0	0
Stauroteuthis syrtensis	✔		82622206	49482062		4003483		50346978			
Sthenoteuthis oualaniensis	✔	1869200834	1869200833	49482116	209970057	1869200831	1869200832	209970223	45510989	45511088	45511042
Stoloteuthis leucoptera	✔	34542064	209969910	49482080	34542113	4003485		50347003			
Octopoteuthis danae<br>**= Taningia danae**	✔	0	209969982	209970029	209970072	209970163	0	209970233	0	0	0
Taonius borealis						5678674					
Taonius pavo	✔	0	209969961	209969992	209970052	209970121	0	209970184	0	0	0
Teuthowenia megalops	✔	48994435	48994458			48762890			48994509	48994609	48994559
Thaumeledone brevis		0	0	0	0	0	0	0	0	0	161611121
Thaumeledone gunteri	✔	161611175	18027462	49482074			161598196	50346993	161611143	158828833	161611123
Thaumeledone peninsulae	✔	161611171	162956983	0	0	0	161598190	0	161611145	158828835	161611125
Thaumeledone rotunda	✔	161611170	161611152				161598186		161611141	158828831	
Thaumeledone sp. CYV 2001		0	0	0	0	15421845	0	0	0	0	0
Thysanoteuthis rhombus	✔	62005901	209969952	209970017	209970046	209970101		209970251			
Todarodes filippovae		0	498955	0	0	0	0	0	0	0	0
Todarodes pacificus	✔	537939954	537939953		18076665	537939951	537939952				
Todaropsis eblanae	✔	48994438	48994462	0	0	0	0	0	48994489	48994615	48994565
Tremoctopus violaceus	✔	45510912	18076192			15421849			45510992	45511052	45511004
Uroteuthis chinensis	✔	0	209969912	23450949	49482138	28207579	0	50347009	0	0	0
Uroteuthis duvauceli			3618168			5678659					
Uroteuthis edulis		0	3618173	0	0	169247791	0	0	0	0	0
Uroteuthis noctiluca	✔	34542041	34542065		34542094	34542116					
Uroteuthis sp. JMS 2004	✔	48762901	48762912	0	0	0	0	0	48994338	48994370	48994400

Appendix 1. GenBank sequence identifiers (GI numbers) for loci used in analysis listed alphabetically. Changes to the species name used in this manuscript are listed in bold. Checkmarks denote taxa used in primary analysis (188-taxa), shown in Fig 1.

GenBank Name	Fig. 1	12S	16S	18S	28S	COI	cytb	H3	odh	opsin	pax
Vampyroteuthis infernalis	✔	1531248574	1531248573	34369180		1531248571	1531248572	50346982	45510945	45511044	45510996
Velodona togata	✔	161611172	161611154	0	0	0	161598184	0	161611147	158828837	161611127
Vitreledonella richardi	✔	45510923	45510943			13249500			45510971	45511074	45511026
Vulcanoctopus hydrothermalis		239735815	239735812	0	0	0	0	0	0	0	0
Watasenia scintillans	✔	893373944	893373943			893373941	893373942				
