# Supplementary material for: A multi-gene phylogeny of Cephalopoda supports convergent morphological evolution in association with multiple habitat shifts in the marine environment
Source: BMC Evol Biol. 2012 Jul 28;12:129. doi: 10.1186/1471-2148-12-129 (PMC3733422; doi:10.1186/1471-2148-12-129)
Supplement: Additional file 3 — Appendix 2.GenBank sequence identifiers (GI numbers) for loci used in analyses listed alphapbetically. Changes to the species name used in this manuscript are listed in bold. Checkmarks denote taxa used in primary analysis (188-taxa), shown in Figure 1. [file 1471-2148-12-129-S3.pdf]

Appendix 2. GI numbers for all contaminant sequences.

Gene	GenBank species name	GenBank GI
12S	Rossia_macrosoma	48994427
	Sepiola_sp_JMS_2004	45510927
16S	Architeuthis_sp_BED_2003	37622176
	Berryteuthis_environmental_sample	184216289
	Berryteuthis_magister	179481868
	Eledone_cirrhosa	16944395
	Eledone_cirrhosa	22384882
	Eledone_cirrhosa	22384880
	Eledone_cirrhosa	22384878
	Eledone_cirrhosa	22384876
	Eledone_cirrhosa	22384874
	Eledone_palari	18073268
	Enteroctopus_dofleini	179481870
	Gonatus_sp_ADS_2008	179481869
	Graneledone_taniwha	18073704
	Loligo_gahi	22384900
	Loligo_gahi	22384898
	Loligo_gahi	22384896
	Loligo_gahi	22384894
	Loligo_gahi	22384892
	Loligo_gahi	22384890
	Loligo_gahi	22384888
	Loligo_opalescens	179481867
	Octopus_vulgaris	22384886
	Pyroteuthis_margaritifera	209969925
	Sepia_latimanus	28207581
	Sepia_officinalis	21842310
	Sepiola_sp_JMS_2004	45510944
	Sepioteuthis_lessoniana	209969913
	Todarodes_sagittatus	21842311
	Uroteuthis_chinensis	14150652
	Uroteuthis_edulis	14150653
18S	Ancistrocheirus_lesueuri	49482096
	Benthoctopus_sp_ARL_2004	49482071
	Heteroteuthis_sp_Spain_WK	42627265
	Histioteuthis_sp_Spain_WK	42627266
	Loligo_vulgaris	42627264
	Sepia_elegans	18026325
	Sepia_elegans	18026324
	Sepia_officinalis	42627260
	Sepietta_sp_France_WK	42627262
	Spirula_spirula	42627259
28S	Benthoctopus_sp_ARL_2004	49482129
	Euprymna_sp	18073642
	Histioteuthis_sp_YJP_2002	34369204
	Idiosepius_pygmaeus	34542093
	Idiosepius_pygmaeus	18073644
	Loligo_forbesi	18073643
	Sepia_officinalis	18076660
	Sepioteuthis_lessoniana	18076666
	Stauroteuthis_syrtensis	82622106

Appendix 2. GI numbers for all contaminant sequences.

Gene	GenBank species name	GenBank GI
CO1	Berryteuthis_magister	58202170
	Berryteuthis_magister	62084189
	Berryteuthis_magister	209970157
	Euprymna_morsei	62084197
	Euprymna_morsei	34542124
	Euprymna_tasmanica	110006704
	Heteroteuthis_dispar	5353816
	Illex_cf_coindetii	87042900
	Illex_cf_coindetii	87042898
	Illex_cf_coindetii	87042890
	Illex_cf_coindetii	87042888
	Illex_cf_coindetii	87042886
	Illex_cf_coindetii	87042884
	Illex_cf_coindetii	87042880
	Illex_cf_oxygonius	87042896
	Illex_cf_oxygonius	87042894
	Illex_cf_oxygonius	87042892
	Illex_cf_oxygonius	87042882
	Illex_cf_oxygonius	87042878
	Illex_cf_oxygonius	87042876
	Rondeletiola_minor	5353818
	Sepia_esculenta	13752531
	Sepia_officinalis	18026441
	Sepia_subtenuipes	242117689
	Thysanoteuthis_rhombus	62084187
cytb	Opisthoteuthis_sp_SA2_MG_2004	76359314
	Rossia_pacifica	237648951
H3	Benthoctopus_sp_ARL_2004	50346989
	Nautilus_scrobiculatus	3002652
	Sepia_officinalis	37654361
odh	Pterygioteuthis_microlampas	48994497
	Sepiola_sp_JMS_2004	45510981
	Sthenoteuthis_oualaniensis	48994499
	Todaropsis_eblanae	48994501
opsin	Rossia_macrosoma	48994589
	Sepiola_sp_JMS_2004	45511082
pax6	Argonauta_nodosa	45511002
	Sepiola_sp_JMS_2004	45511034
