# Supplementary material for: A multi-gene phylogeny of Cephalopoda supports convergent morphological evolution in association with multiple habitat shifts in the marine environment
Source: BMC Evol Biol. 2012 Jul 28;12:129. doi: 10.1186/1471-2148-12-129 (PMC3733422; doi:10.1186/1471-2148-12-129)
Supplement: Additional file 4 — Appendix 3.GI numbers for all contaminant sequences. [file 1471-2148-12-129-S4.pdf]

Appendix 3. Habitat and morphological character data used in analyses. Abbreviations for **Depth range** coded from the literature are: "ukn" = data is not available for a particular species, "?" = a depth could not be defined as no species name was designated, "D" = depth in day, "N" = depth at night, "juv" = depth for juveniles, "OM" = ontogenetic migratory, "VM" = vertical migrator. Habitat states are 0 = pelagic, 1 = benthic/demersal. Character states are (0 = absent, 1 = present) for **ANG, Branchial canal, Autogenic photophore, Bacteriogenic Photophore,** and **Right Oviduct.** States for **Cornea** are: 0 = absent, 1 = 1-part cornea present, 2 = 2-part cornea present.

Species name	Depth Range (m)	Habitat	ANG	Branchial canal	Cornea	Autogenic photophore	Bacteriogenic photophore	Right Oviduct
Abralia veranyi	0 - 800	1	0	1	0	1	0	1
Abraliopsis pacificus	ukn	1	0	1	0	1	0	1
Adelieledone piatkowski	610 - 1510	0	0	1	2	0	0	1
Adelieledone polymorpha	15 - 365	0	0	1	2	0	0	1
Amphioctopus aegina	to 40	0	0	1	2	0	0	1
Ancistroteuthis lichtensteini	0 - 250	1	0	1	0	1	0	1
Architeuthis dux	200 - 800+	1	0	1	0	0	0	1
Architeuthis sp. ARL 2008	200 - 800+	1	0	1	0	0	0	1
Architeuthis sp. JMS 2004	200 - 800+	1	0	1	0	0	0	1
Argonauta nodosus	0 to ?	1	0	1	2	0	0	1
Asperoteuthis nesisi	0 - 1000	1	0	1	0	1	0	1
Bathypolypus arcticus	35 - 1210	0	0	1	2	0	0	1
Bathypolypus sp. JMS 2004	?	0	0	1	2	0	0	1
Bathypolypus valdiviae	200 - 1000	0	0	1	2	0	0	1

Appendix 3 (cont'd)

Species name	Depth Range (m)	Habitat	ANG	Branchial canal	Cornea	Autogenic photophore	Bacteriogenic photophore	Right Oviduct
Bathyteuthis abyssicola	100 - 4200	1	0	1	0	1	0	1
Bathyteuthis berryi	800 -1200+	1	0	1	0	1	0	1
Bathyteuthis sp. A	?	1	0	1	0	1	0	1
Batoteuthis skolops	366 - 2500	1	0	1	0	1	0	1
Benthoctopus eureka	30 - 500	0	0	1	2	0	0	1
Benthoctopus johnsonianus	1850 - 2540	0	0	1	2	0	0	1
Benthoctopus normani	540 - 1835	0	0	1	2	0	0	1
Benthoctopus sp. JMS 2004	?	0	0	1	2	0	0	1
Benthoctopus yaquinae	1000 - 3000	0	0	1	2	0	0	1
Berryteuthis anonychus	0 - 1500	1	0	1	0	0	0	1
Berryteuthis magister	0 - 1500	1	0	1	0	0	0	1
Bolitaena pygmaea	100 - 1000	1	0	1	0	1	0	1
Brachioteuthis sp. 2	?	1	0	1	0	?	0	1
Brachioteuthis sp. 3	?	1	0	1	0	?	0	1
Callistoctopus ornatus	0 - 10	0	0	1	2	0	0	1
Chiroteuthis calyx	0 - 800	1	0	1	0	1	0	1
Chiroteuthis mega	meso- to bathypelagic	1	0	1	0	1	0	1

Appendix 3 (cont'd)

Species name	Depth Range (m)	Habitat	ANG	Branchial canal	Cornea	Autogenic photophore	Bacteriogenic photophore	Right Oviduct
Chiroteuthis veranyi	meso- to bathypelagic	1	0	1	0	1	0	1
Chtenopteryx sicula	meso- to bathypelagic	1	1	1	0	1	0	1
Chtenopteryx sp. ARL 2008	?	1	1	1	0	1	0	1
Cirrothauma murrayi	2430 - 4850	1	0	0	0	0	0	0
Cistopus sp. JMS 2004		0	0	1	0	0	0	1
Cranchia scabra	0 - 2000	1	0	1	0	1	0	1
Cycloteuthis sirventyi	lower epi- to mesopelagic	1	0	1	0	1	0	1
Discoteuthis discus	lower epi- to mesopelagic	1	0	1	0	1	0	1
Discoteuthis laciniosa	ukn	1	0	1	0	1	0	1
Doryteuthis opalescens	0 - 500	0	1	1	1	0	0	0
Doryteuthis pealeii	0 - 400	0	1	1	1	0	0	0
Dosidicus gigas	0 - 1000+	0	0	1	0	1	0	1
Eledone cirrhosa	5 - 500	0	0	1	2	0	0	1
Enoploteuthis higginsi	epi- to mesopelagic	1	0	1	0	1	0	1
Enoploteuthis leptura	epi- to mesopelagic	1	0	1	0	1	0	1
Enteroctopus dofleini	0 - 1500	0	0	1	2	0	0	1
Eucleoteuthis luminosa	ukn	1	0	1	0	1	0	1

Appendix 3 (cont'd)

Species name	Depth Range (m)	Habitat	ANG	Branchial canal	Cornea	Autogenic photophore	Bacteriogenic photophore	Right Oviduct
Euprymna berryi	to 110	0	1	0	1	0	1	0
Euprymna hyllebergi	sp.. not treated	0	1	0	1	0	1	0
Euprymna scolopes	sp.. not treated	0	1	0	1	0	1	0
Euprymna tasmanica	ukn	0	1	0	1	0	1	0
Galiteuthis armata	0 - 2500	1	0	1	0	1	0	1
Galiteuthis sp. JMS 2004	?	1	0	1	0	?	0	1
Gonatopsis octopedatus	0 - 2000	1	0	1	0	0	0	1
Gonatopsis sp. ARL 2008	?	1	0	1	0	0	0	1
Gonatus antarcticus	epi- to bathypelagic	1	0	1	0	0	0	1
Gonatus fabricii	0 - 1000	1	0	1	0	0	0	1
Graneledone antarctica	to 2340	0	0	1	2	0	0	1
Graneledone boreopacifica	1000 - 3000	0	0	1	2	0	0	1
Graneledone verrucosa	850 - 2300	0	0	1	2	0	0	1
Grimalditeuthis bonplandi	ukn	1	0	1	0	1	0	1
Haliphron atlanticus	0 - 3175	1	0	1	2	0	0	1
Hapalochlaena maculosa	0 - 50+	0	0	1	2	0	0	1
Heterololigo bleekeri	0 - 150	0	1	1	1	0	0	0

Appendix 3 (cont'd)

Species name	Depth Range (m)	Habitat	ANG	Branchial canal	Cornea	Autogenic photophore	Bacteriogenic photophore	Right Oviduct
Heteroteuthis hawaiiensis	ukn	1?	1	0	1	0	1	0
Histioteuthis bonellii	500 - 2000	1	0	1	0	1	0	1
Histioteuthis corona	ukn	1	0	1	0	1	0	1
Histioteuthis hoylei	100 - 850	1	0	1	0	1	0	1
Histioteuthis miranda	0 - 1200	1	0	1	0	1	0	1
Histioteuthis oceani	ukn	1	0	1	0	1	0	1
Histioteuthis reversa	0 - 1000	1	0	1	0	1	0	1
Idiosepius notoides	shallow	0	1	0	1	0	0	1 (reduced)
Idiosepius pygmaeus	shallow	0	1	0	1	0	0	1 (reduced)
Illex coindetii	0 - 1000+	1	0	1	0	0	0	1
Japetella diaphana	200 - 1000	1	0	1	0	1	0	1
Joubiniteuthis portieri	D 800-2500, N 300-500	1	0	1	0	0	0	1
Kondakovia sp. ARL 2008	?	1	0	1	0	0	0	1
Leachia atlantica	0 - 1000+	1	0	1	0	1	0	1
Leachia lemur	0 - 1000+	1	0	1	0	1	0	1
Lepidoteuthis grimaldii	0 - 700+	1	0	1	0	0	0	1
Loligo forbesii	50 - 700+	0	1	1	1	0	0	0

Appendix 3 (cont'd)

Species name	Depth Range (m)	Habitat	ANG	Branchial canal	Cornea	Autogenic photophore	Bacteriogenic photophore	Right Oviduct
Loligo vulgaris	0 - 500	0	1	1	1	0	0	0
Loliolus sp. JMS 2004	?	0	1	1	1	0	1?	0
Lolliguncula brevis	shallow water	0	1	1	1	0	0	0
Lolliguncula diomedeae	0 - 200	0	1	1	1	0	0	0
Lycoteuthis lorigera	meso- to bathypelagic	1	0	1	0	1	0	1
Magnapinna sp. ARL 2008	?	1	0	1	0	0	0	1
Mastigoteuthis agassizii	benthopelagic	1	0	1	0	1	0	1
Mastigoteuthis hjorti	benthopelagic	1	0	1	0	1	0	1
Mastigoteuthis magna	benthopelagic	1	0	1	0	1	0	1
Megaleledone setebos	30 - 850	0	0	1	2	0	0	1
Megalocranchia fisheri	ukn	1	0	1	0	1	0	1
Megalocranchia sp. ARL 2008	?	1	0	1	0	1?	0	1
Mesonychoteuthis hamiltoni	20 - 2000	1	0	1	0	1	0	1
Metasepia tullbergi	20 - 100	0	1	0	1	0	0	1
Moroteuthis knipovitchi	0 - 500+	1	0	1	0	0	0	1
Nautilus macromphalus	0 - 500	0	0	0	-	0	0	1
Nautilus pompilius	0 - 750	0	0	0	-	0	0	1

Appendix 3 (cont'd)

Species name	Depth Range (m)	Habitat	ANG	Branchial canal	Cornea	Autogenic photophore	Bacteriogenic photophore	Right Oviduct
Neoteuthis thielei	epi- to benthipelagic; OM	1	0	1	0	0	0	1
Notonykia sp. ARL 2008	?	1	0	1	0	0	0	1
Octopoteuthis megaptera	ukn	1	0	1	0	1	0	1
Octopoteuthis nielseni	ukn	1	0	1	0	1	0	1
Octopoteuthis sicula	epipelagic - 2000	1	0	1	0	1	0	1
Octopus berrima	to 250+	0	0	1	2	0	0	1
Octopus bimaculoides	0 - 20+	0	0	1	2	0	0	1
Octopus cyanea	0 - 20+	0	0	1	2	0	0	1
Octopus kaurna	to 50	0	0	1	2	0	0	1
Octopus ocellatus	?	0	0	1	2	0	0	1
Octopus rubescens	0 - 300	0	0	1	2	0	0	1
Octopus vulgaris	0 - 150	0	0	1	2	0	0	1
Ommastrephes bartramii	0 - 200+	1	0	1	0	0	0	1
Onychoteuthis banksii	0 - 4000	1	0	1	0	1	0	1
Onychoteuthis sp. B3 JMS 2004	?	1	0	1	0	1?	0	1
Onykia carriboea	epipelagic	1	0	1	0	0	0	1
Onykia robusta	0 - 900	1	0	1	0	0	0	1

Appendix 3 (cont'd)

Species name	Depth Range (m)	Habitat	ANG	Branchial canal	Cornea	Autogenic photophore	Bacteriogenic photophore	Right Oviduct
Opisthoteuthis massyae	225 - 1450	1	0	0	0	0	0	0
Ornithoteuthis antillarum	D midwater, N 0-mid depth	1	0	1	0	1	0	1
Pareledone aequipapillae	110 - 465	0	0	1	2	0	0	1
Pareledone albimaculata	190 - 465	0	0	1	2	0	0	1
Pareledone aurata	90 - 465	0	0	1	2	0	0	1
Pareledone charcoti	100 - 700	0	0	1	2	0	0	1
Pareledone cornuta	130 - 455	0	0	1	2	0	0	1
Pareledone panchroma	430 - 805	0	0	1	2	0	0	1
Pareledone serperastrata	130 - 455	0	0	1	2	0	0	1
Pareledone subtilis	190 - 430	0	0	1	2	0	0	1
Pareledone turqueti	0 - 4000	0	0	1	2	0	0	1
Pholidoteuthis adami	350 - 2000	1	0	1	0	0	0	1
Planctoteuthis levimana	0 - 2400	1	0	1	0	0	0	1
Psychroteuthis glacialis	200 - 920	1	0	1	0	1	0	1
Pterygioteuthis gemmata	50 - 800	1	0	1	0	1	0	1
Pterygioteuthis hoylei	0 - 500	1	0	1	0	1	0	1
Pterygioteuthis microlampas	D: 450-500; N: 50-100	1	0	1	0	1	0	1

Appendix 3 (cont'd)

Species name	Depth Range (m)	Habitat	ANG	Branchial canal	Cornea	Autogenic photophore	Bacteriogenic photophore	Right Oviduct
Rondeletiola minor	75 - 495	0	1	0	1	0	1	0
Rossia pacifica	30 - 310/ (550)	0	1	0	1	0	0	0
Rossia palpebrosa	75 - 550	0	1	0	1	0	0	0
Selenoteuthis scintillans	epi- to mesopelagic	1	0	1	0	1	0	1
Sepia apama	0-100	0	1	0	1	0	0	0
Sepia elegans	to 500	0	1	0	1	0	0	0
Sepia esculenta	10 - 100	0	1	0	1	0	0	0
Sepia kobiensis	subtidal - 200	0	1	0	1	0	0	0
Sepia latimanus	to 30	0	1	0	1	0	0	0
Sepia lorigera	100 - 300	0	1	0	1	0	0	0
Sepia lycidas	15 - 100	0	1	0	1	0	0	0
Sepia officinalis	subtidal - 200	0	1	0	1	0	0	0
Sepia pardex	ukn	0	1	0	1	0	0	0
Sepia peterseni	20 - 100	0	1	0	1	0	0	0
Sepia pharaonis	to 130	0	1	0	1	0	0	0
Sepia recurvirostra	10 - 140	0	1	0	1	0	0	0
Sepia sp. SI0604	?	0	1	0	1	0	0	0

Appendix 3 (cont'd)

Species name	Depth Range (m)	Habitat	ANG	Branchial canal	Cornea	Autogenic photophore	Bacteriogenic photophore	Right Oviduct
Sepiadarium austrinum	ukn	0	1	0	1	0	0	0
Sepiadarium kochi	to 60	0	1	0	1	0	0	0
Sepiella inermis	to 40	0	1	0	1	0	0	0
Sepiella japonica	to 50	0	1	0	1	0	0	0
Sepietta neglecta	25 - 475	0	1	0	1	0	0	0
Sepietta obscura	30 - 375	0	1	0	1	0	0	0
Sepiola affinis	20 - 180	0	1	0	1	0	1	0
Sepiola atlantica	epibenthic	0	1	0	1	0	1	0
Sepiola birostrata	to 100	0	1	0	1	0	1	0
Sepiola intermedia	10 - 110/60 - 200	0	1	0	1	0	1	0
Sepiola ligulata	45 - 380	0	1	0	1	0	1	0
Sepiola robusta	25 - 500	0	1	0	1	0	1	0
Sepiolina nipponensis	neritic	0	1	0	1	0	1	0
Sepioloidea lineolata	ukn	0	1	0	1	0	0	0
Sepioteuthis australis	0 - 70	0	1	1	1	0	0	0
Sepioteuthis lessoniana	0 - 100	0	1	1	1	0	0	0
Spirula spirula	D 600-700, N < 300; juv: 1000-1750	1	1	0	0	1	0	0

Appendix 3 (cont'd)

Species name	Depth Range (m)	Habitat	ANG	Branchial canal	Cornea	Autogenic photophore	Bacteriogenic photophore	Right Oviduct
Stauroteuthis gilchristi	to 2560	1	0	0	0	1	0	0
Stauroteuthis syrtensis	500 - 4000	1	0	0	0	1	0	0
Sthenoteuthis oualaniensis	200 - 400+	1	0	1	0	1	0	1
Stoloteuthis leucoptera	160 - 700	1?	0	0	1	0	1	0
Taningia danae	ukn	1	0	1	0	1	0	1
Taonius pavo	200 - 2000	1	0	1	0	1	0	1
Teuthowenia megalops	40 - 2700	1	0	1	0	1	0	1
Thaumeledone gunteri	365 - 965	0	0	1	2	0	0	1
Thaumeledone peninsulae	375 - 1510	0	0	1	2	0	0	1
Thaumeledone rotunda	to 3565	0	0	1	2	0	0	1
Thysanoteuthis rhombus	epi- to mesopelagic	1	0	1	0	0	0	1
Todarodes pacificus	shelf & slope waters	1	0	1	0	0	0	1
Todaropsis eblanae	20 - 250	1	0	1	0	0	0	1
Tremoctopus violaceus	0 - 250	1	0	1	2	0	0	1
Uroteuthis chinensis	10 - 200	0	1	1	1	0	1	0
Uroteuthis noctiluca	0 - 50	0	1	1	1	0	1	0
Uroteuthis sp. JMS 2004	?	0	1	1	1	0	1	0

Appendix 3 (cont'd)

Species name	Depth Range (m)	Habitat	ANG	Branchial canal	Cornea	Autogenic photophore	Bacteriogenic photophore	Right Oviduct
Vampyroteuthis infernalis	600 - 1200	1	0	1	0	1	0	1
Velodona togata	290 - 750	0	0	1	0	0	0	1
Vitreledonella richardi	0 - 1000	1	0	1	0	0	0	1
Watasenia scintillans	100 - 600	1	0	1	0	1	0	1
